# Polydopamine Nanoparticle‐Mediated Dopaminergic Immunoregulation in Colitis

**DOI:** 10.1002/advs.202104006

**Published:** 2021-10-28

**Authors:** Juanjuan Li, Weiliang Hou, Sisi Lin, Lu Wang, Chao Pan, Feng Wu, Jinyao Liu

**Affiliations:** ^1^ State Key Laboratory of Oncogenes and Related Genes Shanghai Cancer Institute Shanghai Key Laboratory for Nucleic Acid Chemistry and Nanomedicine Institute of Molecular Medicine Renji Hospital School of Medicine Shanghai Jiao Tong University Shanghai 200127 China

**Keywords:** dopaminergic, gut microbiota, immunoregulation, inflammatory bowel disease, oral probiotic

## Abstract

Despite immunosuppression is critical for reducing immune overactivation, existing immunosuppressive agents are largely restricted by low inhibition efficiencies and unpredictable off‐target toxicities. Here, the use of the dopaminergic system is reported to suppress hyperactive immune responses in local inflamed tissues. A polydopamine nanoparticular immunosuppressant (PDNI) is synthesized to stimulate regulatory T (Treg) cells and directly inhibit T helper 1 (Th1), Th2, and Th17 cells. Moreover, PDNI can inhibit the activation of dendritic cells to upregulate the ratio of Treg/Th17, which assists the reversion of inflammatory responses. The application of dopaminergic immunoregulation is further disclosed by combining with gut microbiota modulation for treating inflammations. The combination is implemented by coating living beneficial bacteria with PDNI. Following oral delivery, coated bacteria not only suppress the hyperactive immune responses but also positively modulate the gut microbiome in mice characterized with colitis. Strikingly, the combination demonstrates enhanced treatment efficacies in comparison with clinical aminosalicylic acid in two murine models of colitis. The use of the dopaminergic system opens a window to intervene immune responses and provides a versatile platform for the development of new therapeutics for treating inflammatory diseases.

## Graphical abstract

The dopaminergic system is reported to suppress hyperactive immune responses in local inflamed tissues by a polydopamine nanoparticular immunosuppressant, which can stimulate regulatory T lymphocytes and dendritic cells, while directly inhibit T helper cells. The application of dopaminergic immunoregulation is further disclosed by combining with oral probiotics that can modulate the gut microbiota for treating colitis.

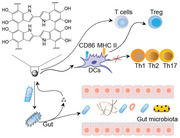

## 1 Introduction

Immunosuppression, an essential way to reduce the overactivation of the immune system,^[^ ^1^ ^]^ is critical for treating inflammations resulted from autoimmune diseases and damage to the immune system.^[^ ^2^, ^3^ ^]^ Small molecular inhibitors of calcineurin, interleukin (IL), and tumor necrosis factor alfa (TNF*α*) are effective to suppress inappropriate inflammatory responses.^[^ ^4^, ^5^ ^]^ Unfortunately, clinical implementations often suffer from limited therapeutic efficacies and systemic side effects,^[^ ^6^, ^7^ ^]^ due to the rapid clearance and lack of specificity that are associated with the characteristics of small molecules.^[^ ^8^, ^9^ ^]^ Though the use of biologics that can inhibit cytokines has been some success, clinical outcomes manifest that inhibition of a single target is often insufficient, yet the toxicity of off‐target inhibition remains unpredictable.^[^ ^10^ ^]^ Engineering cell‐based vehicles and innovating cell derived nanoparticles have emerged as alternatives to directly interact with immune cells^[^ ^11^, ^12^, ^13^, ^14^, ^15^ ^]^ and demonstrated instrumental in treating inflammations, such as arthritis and inflammatory bowel disease (IBD).^[^ ^16^, ^17^, ^18^, ^19^, ^20^ ^]^ However, the utilization of endogenous substances inevitably confronts with potential risks of immunogenicity.

The dopaminergic system is an important part of appropriate immune function.^[^ ^21^ ^]^ Dopamine (DA), which is a catecholamine neurotransmitter, participates in neuroimmune communications and exerts immunomodulatory activity toward various immune cells by dopamine receptors and the related proteins.^[^ ^22^, ^23^ ^]^ For example, DA suppresses activated effector T cells and inhibits their cytokine secretion, proliferation, and other responses and processes.^[^ ^24^ ^]^ Recent studies have revealed that DA mediates the immune functions of the gastrointestinal (GI) tract and maintains gut homeostasis by dopaminergic immunoregulation.^[^ ^25^ ^]^ Low levels of DA promote the expansion of T helper (Th) cells and drive an inflammatory phenotype in CD4^+^ T cells.^[^ ^26^, ^27^ ^]^ DA presents an overall protective characteristic in the gut and precludes the development of intestinal inflammation.^[^ ^28^, ^29^ ^]^ However, it remains unclear how to exploit the dopaminergic system for suppressing hyperactive immune responses, such as inflammations in local pathological tissues.

**Figure 1:**
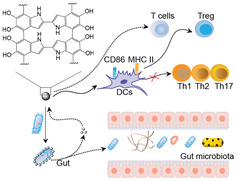
Schematic illustration. Polydopamine nanoparticle‐mediated dopaminergic immunoregulation in inflamed tissue and its combination with the modulation of gut microbiota by coating oral probiotics with PDNI for treating colitis.

Here, we describe the use of dopaminergic immunoregulation to reduce immune overactivation in local inflamed tissues. We synthesize a polydopamine nanoparticular immunosuppressant (PDNI) that carries multiple modalities in immunosuppression by self‐polymerization of DA under an alkaline condition (**Figure** **1**). As a dopamine analogue, PDNI can provoke regulatory T (Treg) cells and directly suppress Th1, Th2, and Th17 cells. In addition to its direct interactions with T cells, PDNI is able to inhibit the activation of dendritic cells (DCs) to upregulate the ratio of Treg/Th17, benefiting the reversion of inflammatory responses in pathological tissues. We further disclose the application of the dopaminergic system for developing potent therapeutics for treating IBD, an intractable disease that affects ≈1.6 million Americans and causes an annual financial burden of more than $30 billion.^[^ ^30^, ^31^ ^]^ We implement dopaminergic immunoregulation by individually coating living probiotic bacteria with PDNI for generating oral biotherapeutics, which not only suppress the hyperactive immune responses but also positively modulate the gut microbiome in mice developed with colitis. Significantly, PDNI decorated bacteria increase the richness and diversity of the gut microbiota and augment the abundance of probiotics, e.g., *Mucispirillum*, a crucial element for repairing intestinal mucosa. On the contrary, pathogens, including *Escherichia_Shigella* and *Proteobacteria* that can induce inflammations, diminish remarkably. Due to the dual effects, the combination of dopaminergic immunoregulation and gut microbiome modulation demonstrates dramatically enhanced therapeutic efficacies in comparison with aminosalicylic acid (ASA), a first‐line drug, in two murine models of colitis. The use of the dopaminergic system provides an avenue for modulating immune responses and offers a universal strategy for the development of unique immunotherapies for treating inflammatory diseases.

## 2 Results and Discussion

### 2.1 Characterization of PDNI

**Figure 2:**
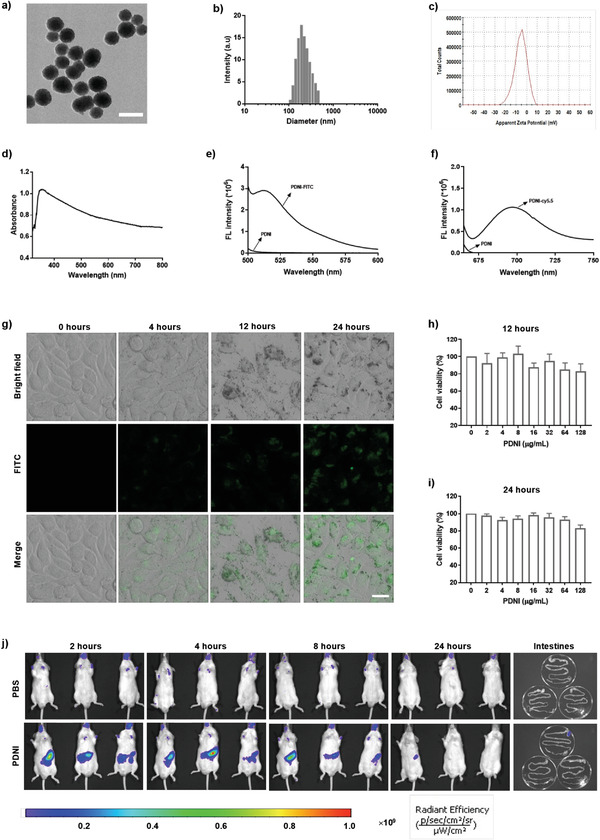
Characterization of PDNI in vitro and in vivo. a) A representative TEM image of PDNI. Scale bar: 200 nm. DLS measurements of b) hydrodynamic size distribution and c) zeta potential of PDNI. d) UV–vis spectrum of PDNI. Fluorescence spectra of PDNI labeled with e) FITC and f) cy5.5, respectively. g) CLSM images of MODE‐K cells after incubation with PDNI‐FITC (100 µg mL^−1^) for different time intervals. Scale bar: 20 µm. Cell viability of MODE‐K cells after culture with PDNI for h) 12 and i) 24 h, respectively. j) In vivo imaging system measurement of mice after oral administration with PDNI‐cy5.5 (100 mg kg^−1^) for different time points. Fluorescence images of intestines were recorded at 24 h postgavage.

We prepared PDNI by self‐polymerization of DA in Tris‐HCl (pH 8.8). Transmission electron microscopy (TEM) observation of PDNI presented a spherical morphology (**Figure** **2**a). Dynamic light scattering (DLS) measurement showed that PDNI had an average diameter around 150 nm (polydispersity index (PDI) = 0.203) (Figure 2b) and a zeta potential of −5.5 mV (Figure 2c). Ultraviolet‐visible (UV–vis) spectrum of PDNI exhibited a characteristic band around 380 nm, which demonstrated the generation of polydopamine (Figure 2d). To investigate the interaction between PDNI and cells, we labeled PDNI with fluorescein isothiocyanate (FITC).^[^ ^32^, ^33^ ^]^ An emission peak at 525 nm appeared at the spectrum of PDNI‐FITC indicated that PDNI were successfully labeled with FITC (Figure 2e). Both MODE‐K and Caco‐2 cell lines were selected to evaluate the cytotoxicity of PDNI.^[^ ^34^ ^]^ Confocal laser scanning microscopy (CLSM) images showed that PDNI could be easily internalized by these cells (Figure 2g and Figure S1, Supporting Information). As expected, PDNI exhibited limited cytotoxicity against MODE‐K cells even with concentration increasing up to 125 µg mL^−1^ (Figure 2h,i). To study the metabolism of PDNI in vivo, we marked PDNI with cy5.5 (Figure 2f) and then orally administered mice with 100 mg kg^−1^ of PDNI‐cy5.5. In vivo imaging system measurement indicated that the dosed mice showed highest fluorescence intensity at 4 h postadministration, which decreased gradually and almost disappeared with observation extending to 24 h (Figure 2j). Similar results were observed by detecting the levels of PDNI in the fecal samples (Figure S2a, Supporting Information), suggesting that PDNI could be cleaned efficiently by gastrointestinal passage. Furthermore, there was no fluorescence signal detected in the major organs of the treated mice (Figure S2b, Supporting Information). Hematoxylin and eosin (H&E) staining of representative colon, liver, and spleen tissues sampled from the treated mice displayed no significant difference in comparison with those sectioned from untreated mice (Figure S2c, Supporting Information), verifying satisfactory compatibility of PDNI for oral administration.

### 2.2 Immunomodulation Activity of PDNI in Inflamed Tissues

**Figure 3:**
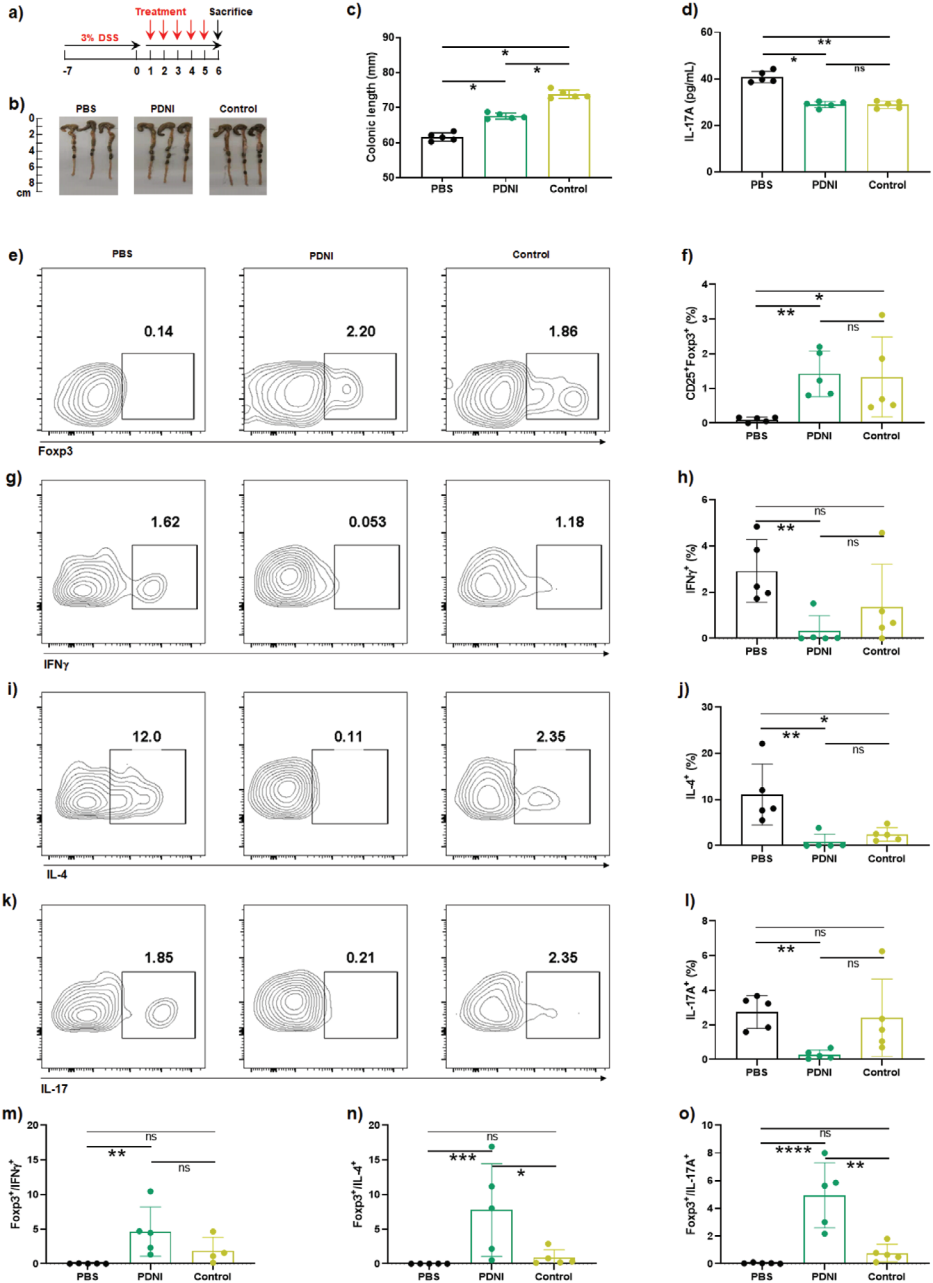
Immunomodulation activity of PDNI in the inflamed tissue. a) Mice were pretreated with 3% DSS drinking water for a week and then orally dosed with PDNI (10 mg kg^−1^) by gavage. Mice without pretreatment were used as a control. LPMC were harvested from the inflamed colon 5 d postadministration and immediately marked with corresponding antibodies after stimulation with phorbol 12‐myristate 13‐acetate (PMA) (10 ng mL^−1^), ionomycin (1 µg mL^−1^), and Brefeldin A (10 µg mL^−1^) for 6 h. b) Photographs of colons sectioned from the treated mice. c) Variations of colon length after treatment. d) Level of IL‐17A in serum sampled from the treated mice. Flow cytometric analysis of LPMC marked with e) anti‐CD4‐FITC/anti‐CD25‐PE‐cy7/anti‐Foxp3‐PE, g) anti‐CD4‐FITC/anti‐IFN*γ*‐PE‐cy7, i) anti‐CD4‐FITC/anti‐IL‐4‐APC, and k) anti‐CD4‐FITC/anti‐IL‐17A‐PE. Cells were pregated on CD3^+^ cells. Percentages of f) CD4^+^CD25^+^Foxp3^+^, h) CD4^+^IFN*γ* ^+^, j) CD4^+^IL‐4^+^, and l) CD4^+^IL‐17A^+^ cells in LPMC, respectively. Ratios of m) CD4^+^CD25^+^Foxp3^+^/CD4^+^IFN*γ* ^+^, n) CD4^+^CD25^+^Foxp3^+^/CD4^+^IL‐4^+^, and o) CD4^+^CD25^+^Foxp3^+^/CD4^+^IL‐17A^+^ in LPMC. Error bars represent standard error of mean (*n* = 5). *p* < 0.05, *, *p* < 0.01, **, *p* < 0.001, ***. ns indicates no statistical significance.

We then tested the immunomodulatory activity of PDNI on lamina propria mononuclear cells (LPMC) from inflamed colon developed by giving mice with 3% dextran sulfate sodium (DSS) in drinking water.^[^ ^35^ ^]^ The abundances of Treg, Th1, Th2, and Th17 cells in LPMC were detected considering that their imbalance was linked with the progress of inflammation.^[^ ^36^ ^]^ Mice without pretreatment with DSS and DSS mice dosed with phosphate buffered saline (PBS) were used as controls (**Figure** **3**a). Apparently, PDNI increased the colonic length of the treated mice (Figure 3b,c). LPMC were marked with anti‐CD4‐FITC/anti‐CD25‐phycoerythrin (PE)‐cy7/anti‐Foxp3‐PE or anti‐CD4‐FITC/anti‐interferon *γ* (IFN*γ*)‐PE‐cy7/anti‐IL‐4‐allophycocyanin (APC)/anti‐IL‐17A‐PE to classify Treg, Th1, Th2, and Th17 cells (Figure 3e,g,i,k). Significantly, PDNI upregulated the corresponding CD4^+^CD25^+^Foxp3^+^ cells (Figure 3f) and decreased the percentages of CD4^+^IFN*γ* ^+^, CD4^+^IL‐4^+^, and CD4^+^IL‐17A^+^ cells in LPMC (Figure 3h,j,l). Furthermore, the use of PDNI increased the ratios of Treg/Th1, Treg/Th2, and Treg/Th17 (Figure 3m–o), which recovered to the same level of uninfected mice, indicating the effect of PDNI on antiinflammation. The concentration of IL‐17A in serum from mice dosed with PDNI reduced as a result of upgrading Treg/Th17, whereas IL‐10 showed no difference comparing to mice treated with PBS (Figure 3d and Figure S3b, Supporting Information). In addition, PDNI reduced the level of tumor growth factor *β* (TGF*β*) in comparison with mice in the PBS group (Figure S3a, Supporting Information). We further developed a CD4^+^ T cell dependent colitis model by administering with 2.5% 2,4,6‐trinitrobenzenesulfonic acid (TNBS) through anus following pretreatment with 1% TNBS on their back (Figure S4a, Supporting Information). PDNI remarkably improved the ratios of Treg/Th1, Treg/Th2 and Treg/Th17 in LPMC (Figure S4b–d, Supporting Information), indicating that PDNI could ameliorate the inflammation by regulating Treg, Th1, Th2, and Th17 cells.

### 2.3 Immunomodulation Ability of PDNI on DCs and T Lymphocytes

**Figure 4:**
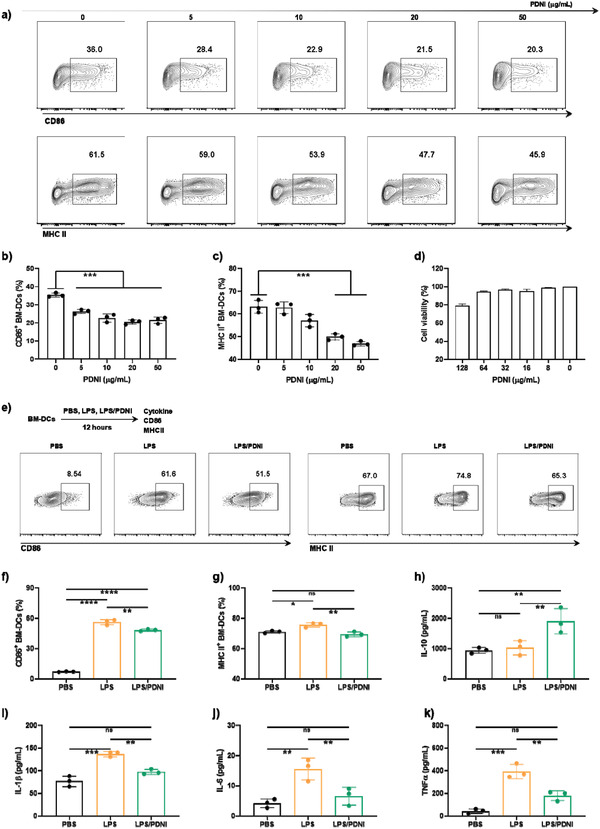
Immunomodulation ability of PDNI on BM‐DCs. a) Flow cytometric analysis of BM‐DCs after incubation with different concentrations of PDNI for 12 h. Cells were marked with anti‐MHC II‐PE and anti‐CD86‐APC. Cells were pregated on CD11c^+^ cells. Percentages of b) CD86^+^ and c) MHC II^+^ DCs after incubation with different concentrations of PDNI for 12 h. d) Cell viability of BM‐DCs after culture with PDNI for 12 h. e) Flow cytometric analysis of BM‐DCs after cultivation with PBS, LPS, and LPS/PDNI for 12 h, respectively. Cells were marked with anti‐MHC II‐PE and anti‐CD86‐APC. Cells were pregated on CD11c^+^ cells. Expressions of f) CD86 and g) MHC II on BM‐DCs after incubation with PBS, LPS, and LPS/PDNI for 12 h, respectively. Levels of h) IL‐10, i) IL‐1*β*, j) IL‐6, and k) TNF*α* in the culture media after different treatments. Error bars represent standard error of mean (*n* = 3). *p* < 0.05, *, *p* < 0.01, **, *p* < 0.001, ***, *p* < 0.0001, ****. ns indicates no statistical significance.

To understand the activity toward LPMC in the inflamed tissue, we studied the immunomodulation ability of PDNI on bone marrow DCs (BM‐DCs) and CD4^+^ lymphocytes. To illuminate the immune activity, we evaluated the effect of PDNI on the expressions of CD86 and major histocompatibility complex (MHC) II on BM‐DCs, as which were responsible for presenting antigens to CD4^+^ lymphocytes. Cell viability of BM‐DCs after incubation with PDNI for 12 h indicated the low cytotoxicity toward BM‐DCs even with the concentration of PDNI increasing up to 125 µg mL^−1^ (**Figure** **4**d). PDNI decreased the expressions of both CD86 and MHC II on BM‐DCs after incubation (Figure 4a–c), which could inhibit the mature and antigen presentation of BM‐DCs. We further investigated the immunosuppression ability of PDNI toward BM‐DCs that were activated by lipopolysaccharides (LPS).^[^ ^37^ ^]^ Cells were treated with PBS, LPS, and LPS/PDNI, respectively. Effectively, PDNI prohibited the activation of LPS on BM‐DCs, as reflected by the decreased expressions of CD86 and MHC II (Figure 4e–g). Moreover, PDNI raised the level of IL‐10, while decreased the concentrations of IL‐1*β*, IL‐6, and TNF*α* (Figure 4h–k), showing its capability to lower the expression of MHC II and prohibit the activation of inflammatory cells.^[^ ^38^ ^]^

**Figure 5:**
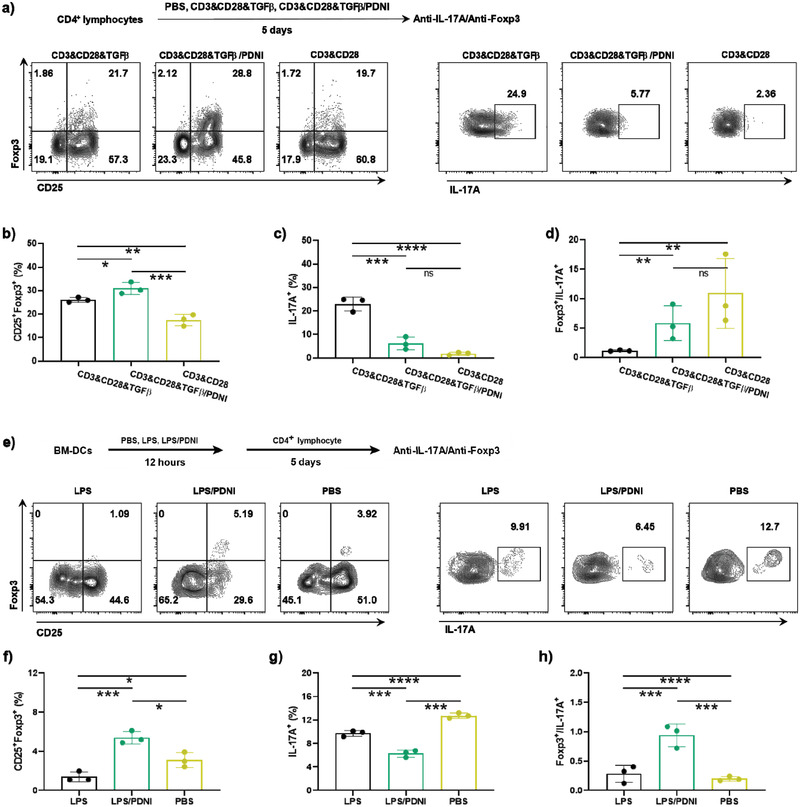
Immunomodulation ability of PDNI on CD4^+^ lymphocytes. a) Flow cytometric analysis of CD4^+^ lymphocytes after cultivation with CD3&CD28, CD3&CD28&TGF*β*, and CD3&CD28&TGF*β*/PDNI for 5 d, respectively. Cells were marked with anti‐CD3‐Percp‐cy5.5, anti‐CD4‐FITC, anti‐CD25‐PE‐cy7, anti‐IL‐17A‐PE, and anti‐Foxp3‐APC. Cells were pregated on CD3^+^ cells. Percentages of b) CD25^+^Foxp3^+^ cells and c) IL‐17A^+^ in CD4^+^ lymphocytes after incubation with CD3&CD28, CD3&CD28&TGF*β*, and CD3&CD28&TGF*β*/PDNI, respectively. d) Ratio of Foxp3^+^/IL‐17A^+^ cells. e) Flow cytometric analysis of CD4^+^ lymphocytes after incubation with BM‐DCs pretreated with PBS, LPS, and LPS/PDNI for 12 h, respectively. Cells were pregated on CD3^+^ cells. Percentages of f) CD25^+^Foxp3^+^ cells and g) IL‐17A^+^ in CD4^+^ lymphocytes after incubation with pretreated BM‐DCs. h) Ratio of Foxp3^+^/IL‐17A^+^ in lymphocytes. Error bars represent standard error of mean (*n* = 3). *p* < 0.05, *, *p* < 0.01, **, *p* < 0.001, ***, *p* < 0.0001, ****. ns indicates no statistical significance.

We then studied the ability of PDNI to regulate the immune balance between Treg and Th17 in CD4^+^ lymphocytes. We treated CD4^+^ lymphocytes with anti‐CD3&CD28, anti‐CD3&CD28&TGF*β*, and anti‐CD3&CD28&TGF*β*/PDNI for 5 d respectively and then marked with anti‐CD25‐PE‐cy7, anti‐CD4‐FITC, anti‐IL‐17A‐PE, or anti‐Foxp3‐APC (**Figure** **5**a). PDNI was able to increase the percentage of CD25^+^Foxp3^+^ cells and decrease the percentage of IL‐17A^+^ cells in CD4^+^ lymphocytes stimulated by anti‐CD3&CD28&TGF*β* (Figure 5b,c). Correspondingly, PDNI elevated the ratio of Treg/Th17 (Figure 5d), which could suppress the immune overreaction in inflammatory sites.^[^ ^39^ ^]^ We further cocultured CD4^+^ lymphocytes with BM‐DCs that separately pretreated with PBS, LPS, and LPS/PDNI. As shown in Figure 5e–h, LPS/PDNI pretreated BM‐DCs not only diminished the percentage of IL‐17A^+^ cells but also increased the ratio of CD25^+^Foxp3^+^ cells, in comparison with those of LPS pretreated BM‐DCs. As expected, coculturing with LPS/PDNI pretreated BM‐DCs led to an increase in Foxp3^+^/IL‐17A^+^, indicating that PDNI upregulated the ratio of Treg/Th17 in CD4^+^ lymphocytes. Taken together, PDNI directly reversed the stimulation of T lymphocytes into Th17 and increased the percentage of Treg by decreasing the expression of MHC II on DCs. The immunomodulation on both T lymphocytes and DCs could be attributed to the degradation of PDNI, releasing a small fraction of DA and other fragments of polydopamine.^[^ ^40^ ^]^ While, it was worth mentioning that direct oral administration of free DA might cause potential side effects, although dopamine presented benefits in the modulation of inflammation.

### 2.4 Implementation of Dopaminergic Immunoregulation by Combining with Gut Microbiota Modulation

IBD involves an inappropriate immune response to the intestinal tract triggered by microorganism invaders or antigens, though the exact cause remains elusive. Conventional immunosuppressants can modulate the activity of the immune system and retard ongoing inflammation. However, their implementations are restrained to maintaining remission in patients who have not responded to other therapeutics or who have only responded to steroids.^[^ ^41^ ^]^ On the other hand, the advances within the field of the gut microbiome have provided targets for the development of effective therapies. The incidence of IBD is accompanied by dysbiosis and loss of taxonomic diversity, which lead to the accumulation and translocation of pathogens. Increasing the abundance of probiotic bacteria in the microflora is of great benefit for treatment. We hence combined the immunoregulation with gut microbiota modulation to disclose the potential of implementing the dopaminergic system.

**Figure 6:**
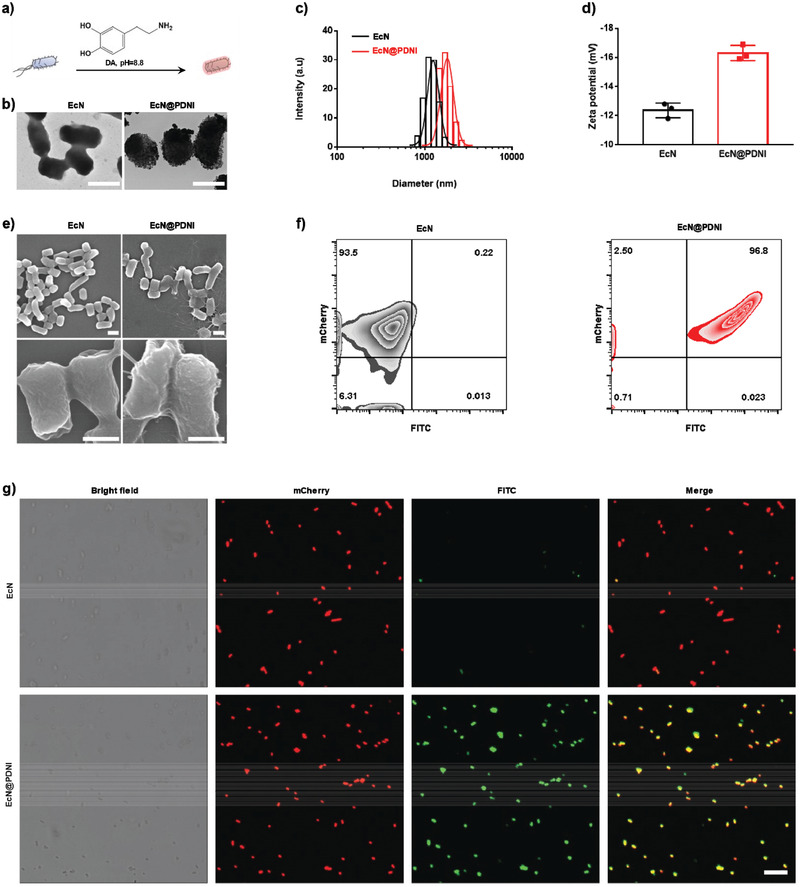
Characterization of EcN@PDNI. a) Schematic illustration of coating living bacteria with PDNI. b) Typical TEM images of EcN and EcN@PDNI. Scale bar: 1 µm. 10 µL of the bacterial solution was loaded onto a 300‐mesh copper formvar/carbon grid and rinsed twice by double distilled water. All samples were observed immediately after completely dried in air. DLS measurements of c) size distribution and d) zeta potential of EcN and EcN@PDNI. Error bars represent standard error of mean (*n* = 3). *p* < 0.01, **. e) Representative SEM images of EcN and EcN@PDNI. Scale bar: 1 µm (top) and 0.5 µm (bottom). The bacteria were fixed by glutaraldehyde and dehydrated serially by ethanol. Samples were lyophilized and coated with the help of a sputtering coater just before observation. f) Flow cytometric profile of EcN@PDNI after labeling PDNI with FITC. g) CLSM images of mCherry expressing EcN@PDNI after labeling PDNI with FITC. Scale bar: 10 µm.

We coated living probiotic bacteria individually with PDNI given the adhesion behavior of polyphenol structure. As a gram‐negative bacterium, EcN have also been reported to be able to interact with dopamine.^[^ ^42^ ^]^ Coating bacteria with PDNI could not only facilitate the delivery of PDNI into the intestine via a single oral dose but also protected the coated bacteria from gastric acid in the stomach and the bile acids in the duodenum. As a proof‐of‐concept study, we chose probiotic *Escherichia coli Nissle 1917* (EcN), a well‐known gut bacterium, which has demonstrated effective for diagnosis and treatment in our previous studies.^[^ ^43^, ^44^ ^]^ PDNI‐coated EcN (EcN@PDNI) were prepared by shaking the bacteria with DA in Tri‐HCL (pH 8.8) for 0.5 h at room temperature (**Figure** **6**a). The density of PDNI appeared on EcN increased with DA concentration (Figure S5, Supporting Information), suggesting the self‐polymerization of DA on the bacteria. TEM images showed that incubating with 1 mg mL^−1^ of DA generated an entire coating composed by polydopamine nanoparticles (Figure 6b). Coating with PDNI enlarged the hydrated size of the bacteria from 1200 to 1750 nm (Figure 6c). Oppositely, the zeta potential decreased from −12.4 ± 0.5 to −16.3 ± 0.4 mV (Figure 6d). Scanning electron microscopy (SEM) images displayed a relative smooth surface for EcN, which turned to much rough after PDNI decoration (Figure 6e). Flow cytometric analysis suggested that the intensity of fluorescence enhanced largely after decorating with FITC‐labeled PDNI (Figure 6f). In contrast to unmodified EcN, the overlap of fluorescent signals between FITC‐labeled PDNI and EcN expressing mCherry in the CLSM images of EcN@PDNI further verified the self‐polymerization of DA on the bacteria (Figure 6g). In vitro assay of EcN@PDNI displayed limited cytotoxicity against MODE‐K cells after 24 h incubation (Figure S6a,b, Supporting Information). Furthermore, EcN@PDNI could bind onto cell surface rather than enter into cells due to the characteristic of an extracellular strain (Figure S6c, Supporting Information).

### 2.5 Viability and Structural Stability of EcN@PDNI

**Figure 7:**
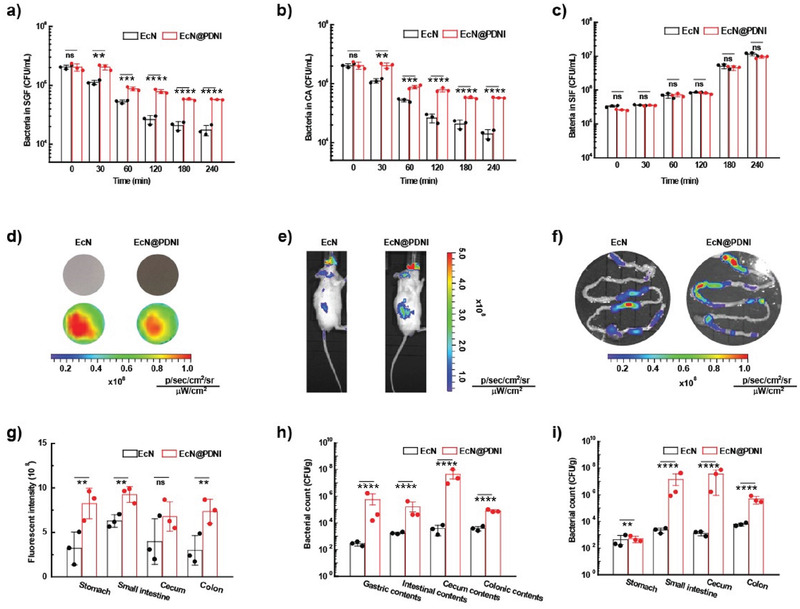
Viability and structural stability of EcN@PDNI. Viabilities of bacteria after exposure to a) SGF, b) 0.3 mg mL^−1^ CA, and c) SIF for the indicated time points. d) Fluorescent images of the same amount of EcN and EcN@PDNI expressing mCherry. e) Typical images captured by in vivo imaging system 4 h postadministration of an oral dose of 2 × 10^8^ CFU EcN or EcN@PDNI. f) Representative fluorescent images of the GI tract sectioned from the dosed mice. g) Quantification of the fluorescence intensities of the stomach, small intestine, cecum, and colon by in vivo imaging system. Counts of bacteria colonized in h) tissues and i) the associated contents of the stomach, small intestine, cecum and colon, respectively. Samples were homogenized, spread onto ager plates and cultured at 37 °C for 24 h before bacterial counting. Error bars represent standard error of mean (*n* = 3). *p* < 0.01, **, *p* < 0.001, ***, *p* < 0.0001, ****. ns indicates no statistical significance.

To further understand the relationship between the bioactivity and structure of EcN@PDNI, we studied the bacterial viability and the stability of PDNI coating, which we speculated were associated with the treatment efficacy. We evaluated the viabilities of EcN@PDNI against simulated gastric fluid (SGF) and 0.3 mg mL^−1^ cholic acid (CA) given that gastric acid and bile acids could deactivate bacteria after oral administration.^[^ ^45^ ^]^ Interestingly, EcN@PDNI displayed strengthened viabilities, which were five to ten times higher than those of uncoated bacteria (**Figure** **7**a,b and Figures S7–S9, Supporting Information). Uncoated bacteria damaged severely after 4 h exposure to SGF, whereas EcN@PDNI remained their initial morphology (Figure S10, Supporting Information). As verified by the similar growth profile to uncoated bacteria, EcN@PDNI proliferated normally in simulated intestinal fluid (SIF) (Figure 7c and Figures S11 and S12, Supporting Information), demonstrating that PDNI protected the bacteria from environmental insults, also had ignorable influence on their proliferation. We further monitored the stability of PDNI coating by incubating EcN@PDNI in SIF and found that the fluorescence signal from FITC‐labeled PDNI decreased with bacterial growth (Figure S13a,b, Supporting Information). Meantime, the zeta potential of the bacteria increased from −15.5 to −12.5 mV and the average size decreased from 2 to 1.3 µm (Figure S13c,d, Supporting Information). Additionally, the supernate of EcN@PDNI cultivated in SIF showed increasing absorbance at 280 nm, further indicating the decoating of PDNI (Figure S14, Supporting Information). The distribution evaluation revealed uninfluenced motility of EcN@PDNI in contrast to that of bare EcN (Figure S15a, Supporting Information).

We next explored the in vivo fate of EcN@PDNI following oral administration. Fluorescence images captured by in vivo imaging system manifested that the bacteria showed similar fluorescence intensity after decorating with PDNI (Figure 7d). Mice dosed with EcN@PDNI had a higher fluorescence intensity than that administrated with uncoated bacteria (Figure 7e). The enhanced signals were further observed for the sectioned GI tract 4 h postadministration (Figure 7f,g). To quantify the survival of EcN@PDNI in the gut, both tissues and the associated contents from the stomach, small intestine, cecum, and colon were harvested and homogenized for bacterial plate counting. Greatly enhanced survivals were found for EcN@PDNI in all these locations (Figure 7h,i), which might be ascribed to the adhesive effect of PDNI.^[^ ^46^ ^]^ The numbers of EcN increased by a couple of orders of magnitude by decorating with PDNI. Additionally, EcN extended the retention time of the surface PDNI in the intestine compared to free PDNI (Figure S15b, Supporting Information). These results illustrated that in addition to its immunoregulation, PDNI could protect EcN from the GI tract stressors and consequently improve their survival in vivo.

### 2.6 Treatment Efficacy of EcN@PDNI in IBD Mice

**Figure 8:**
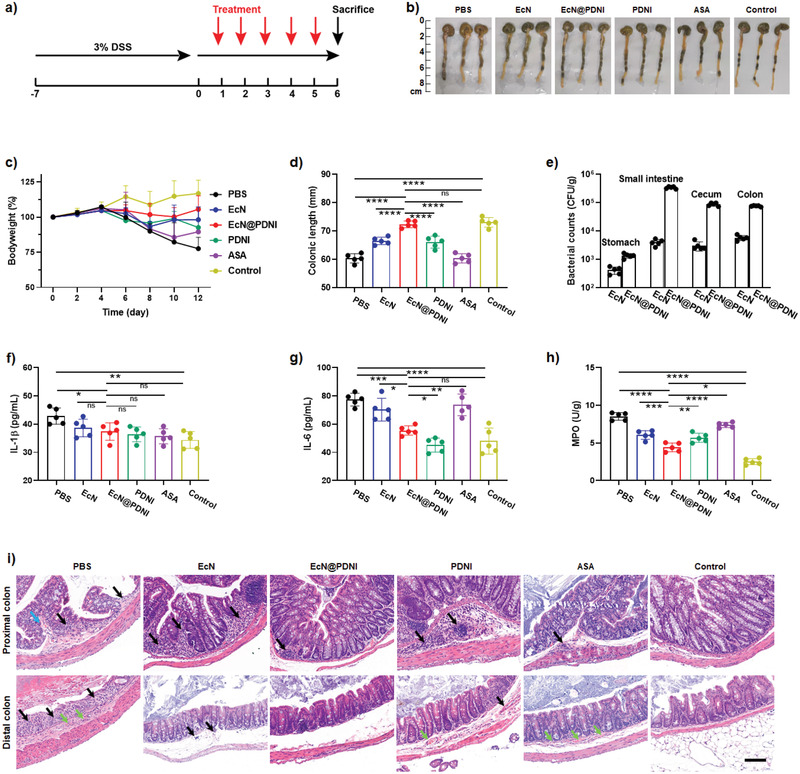
Treatment efficacy of EcN@PDNI in DSS mice. a) Experimental design for treating a DSS induced murine model of colitis. Mice were pretreated with 3% DSS drinking water for a week to develop colitis and then orally dosed with 0.2 mL PBS, EcN (2 × 10^8^ CFU), EcN@PDNI (2 × 10^8^ CFU), PDNI (10 mg kg^−1^), and ASA (60 mg kg^−1^) by gavage, respectively. All mice were euthanatized for sampling 5 d post‐treatment. Mice without pretreatment were used as a control. b) Photographs of colons sectioned from the treated mice. c) Variation of bodyweight after treatment. d) Lengths of colons after treatment. e) Numbers of EcN colonized in the stomach, small intestine, cecum, and colon. 1 cm of each tissue was sampled and homogenized for bacterial plate counting. Expression levels of f) IL‐1*β* and g) IL‐6 in serum collected from mice in each group. h) Levels of MPO in colons after treatment. i) Typical H&E staining of proximal and distal colons. Black, blue, and green arrows indicate inflammation, hemorrhage, and edema, respectively. Scale bar: 50 µm. Error bars represent standard error of mean (*n* = 5). *p* < 0.05, *, *p* < 0.01, **, *p* < 0.001, ***, *p* < 0.0001, ****. ns indicates no statistical significance.

We examined the therapeutic efficacy of EcN@PDNI against DSS‐induced colitis. ASA, a first‐line drug, was used as a control.^[^ ^47^ ^]^ Moreover, uninfected mice and DSS mice dosed with PBS were exploited as controls. It was worth noting that the characteristics of colitis in mice induced by DSS were similar to that in humans, including bodyweight loss, bloody diarrhea, ulcer formation, and loss of epithelial cells.^[^ ^48^, ^49^ ^]^ To assess the therapeutic effect, mice were treated with consecutive five oral doses of 2 × 10^8^ Colony‐Forming Unit (CFU) EcN, EcN@PDNI (2 × 10^8^ CFU), PDNI (10 mg kg^−1^), and ASA (60 mg kg^−1^) without antibiotic pretreatment, as which could disrupt the homeostasis of the gut microbiota. Mice were euthanatized 5 d postadministration (**Figure** **8**a). We found that only treatment with EcN@PDNI could effectively recover the normal patterns of bodyweight and stool for DSS mice (Figure 8c). Treating with EcN@PDNI displayed similar colonic length to that of uninfected mice, which was separately 26, 10, 12, and 19 mm longer than those of mice administrated with PBS, EcN, PDNI, and ASA (Figure 8b–d). The dosed bacteria were able to colonize inside the GI tract, with ≈10^5^ CFU per centimeter of the small intestine, cecum, and colon, respectively (Figure 8e), which was tenfold higher than that of mice fed with EcN. In comparison with mice dosed with PBS, EcN@PDNI reduced the level of IL‐1*β* in serum, which approximated to those of uninfected mice (Figure 8f). Furthermore, mice administered with EcN@PDNI presented the lowest concentration of IL‐6 in all treated mice, demonstrating the ability to relieve the inflammatory responses in DSS mice (Figure 8g). The level of myeloperoxidase (MPO) in the colon of mice treated with EcN@PDNI also showed a remarkable decrease in contrast to all treated mice (Figure 8h). Pathological examination of proximal and distal colon revealed that the loss of epithelial cells happened to PBS treated mice (Figure 8i). The remained inflammation in the proximal colon could be explained by that the damage to mucosal layer was not recovered completely after treatment. Treatments with EcN@PDNI and PDNI showed limited variation on the concentration of serum serotonin (Figure S16a, Supporting Information). It was noted that mice after PDNI and EcN@PDNI treatments appeared elevated food intake and action activity. DSS induced inflammation, hemorrhage, and edema were also observed for PBS treated mice. Despite the administration of EcN, PDNI, or ASA delivered beneficial effect, the use of EcN@PDNI eliminated the inflammation and reduced the hemorrhage and edema most efficiently, which were validated by the lowest histopathology score (mean score 0.6) (Figure S16b, Supporting Information). These results suggested the enhanced therapeutic efficacy of EcN@PDNI, as verified by the rehabilitated colon, normal levels of inflammatory cytokines and the restored pathology.

### 2.7 Immunoregulation and Gut Microbiota Modulation in IBD Mice

**Figure 9:**
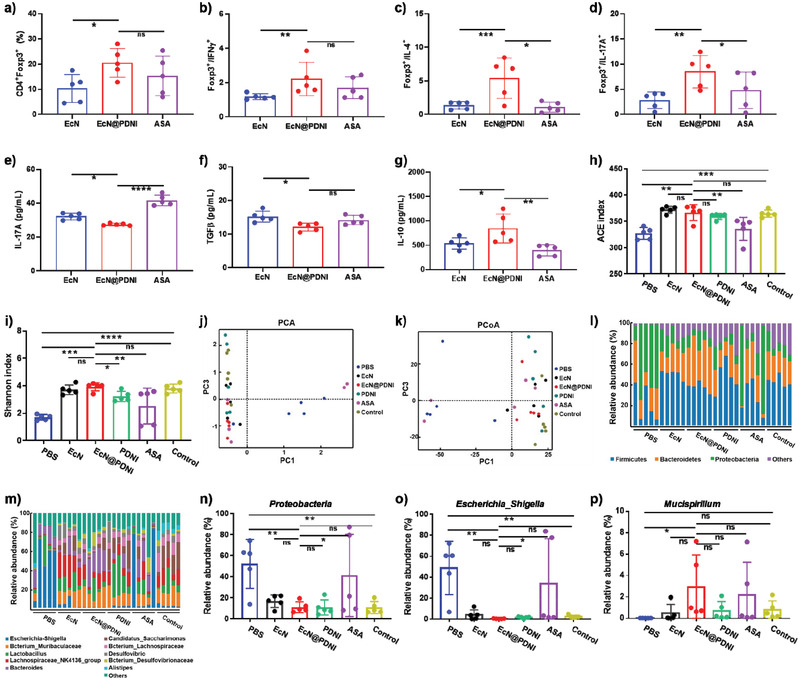
Immunomodulation activity and modulation of the gut microflora by EcN@PDNI in IBD mice. Mice with DSS induced colitis were daily dosed with 0.2 mL PBS, EcN (2 × 10^8^ CFU), EcN@PDNI (2 × 10^8^ CFU), PDNI (10 mg kg^−1^), and ASA (60 mg kg^−1^) for 5 d and then euthanatized for sampling. Mice without DSS induction were used as a control. a) Percentages of CD4^+^CD25^+^Foxp3^+^ cells in LPMC. Ratios of b) CD4^+^CD25^+^Foxp3^+^/CD4^+^IFN*γ* ^+^, c) CD4^+^CD25^+^Foxp3^+^/CD4^+^IL‐4^+^, and d) CD4^+^CD25^+^Foxp3^+^/CD4^+^IL‐17A^+^ in LPMC. Expression levels of e) IL‐17A, f) TGF*β*, and g) IL‐10 in serum sampled from the treated mice. Values of h) ACE and i) Shannon index of the gut microflora. Large values of ACE and Shannon index reflect high richness and evenness of the gut microflora, respectively. Results of j) PCA and k) PCoA of the gut microflora. Short distances of groups indicate small difference among groups. Abundances of l) phyla and m) genus in the gut microflora. Abundances of n) *Proteobacteria*, o) *Escherichia_Shigella*, and p) *Mucispirillum* in the gut microflora. Both *Proteobacteria* and *Escherichia_Shigella* are pathogens, which increase once colitis happens. *Mucispirillum* assists repairing intestinal mucosa by colonizing in colon. Error bars represent standard error of mean (*n* = 5). *p* < 0.05, *, *p* < 0.01, **, *p* < 0.001, ***, *p* < 0.0001, ****. ns indicates no statistical significance.

We speculated that the therapeutic effect of EcN@PDNI was attributed to their abilities to suppress the immune overreaction in the inflamed colon and positively modulate the gut microbiota.^[^ ^50^ ^]^ To prove this hypothesis, we first analyzed the abundances of Treg, Th1, Th2, and Th17 cells in LPMC sampled from DSS mice treated with EcN, EcN@PDNI, and ASA, respectively. The harvested LPMC were similarly marked with anti‐CD4‐FITC/CD25‐PE‐cy7/anti‐Foxp3‐PE or anti‐CD3‐Percp‐cy5.5/anti‐CD4‐FITC/anti‐IFN*γ*‐PE‐cy7/anti‐IL‐4‐APC/anti‐IL‐17A‐PE to classify Treg, Th1, Th2, and Th17, respectively. LPMC from mice treated with EcN and ASA were used as controls. Comparing to EcN and ASA, the implementation of EcN@PDNI lifted the percentage of CD4^+^Foxp3^+^ cells (**Figure** **9**a) as well as the ratios of Treg/Th1, Treg/Th2, and Treg/Th17 in LPMC (Figure 9b–d). Furthermore, treating with EcN@PDNI not only lowered the level of IL‐17A but also increased the level of IL‐10 (Figure 9e,g). Comparing to EcN dosed mice, decreased level of TGF*β* was found to EcN@PDNI (Figure 9f). We further analyzed the levels of CD86 and MHC II on DCs. As shown in Figure S17 in the Supporting Information, a significant decrease of MHC II expression was presented, despite no significance was observed to CD86. Similar to PDNI, EcN@PDNI showed dopaminergic immunoregulation activity in IBD mice.

We next investigated the variation of the microbiome after treating with EcN@PDNI, to understand whether the therapeutic effect was resulted from the combination of dopaminergic immunoregulation and gut microbiota modulation. We analyzed the microflora of DSS mice post consecutive five oral doses of EcN, EcN@PDNI, PDNI, and ASA, respectively. Significantly, EcN@PDNI augmented the richness and evenness of the gut microflora, as indicated by the high values of abundance‐based coverage estimator (ACE) and Shannon index (Figure 9h,i). Both principal component analysis (PCA) and principal coordinates analysis (PCoA) suggested that EcN@PDNI treated mice exposed notable difference in the microbiome in comparison with mice applied with PBS, EcN, PDNI, and ASA, respectively (Figure 9j,k). EcN@PDNI dramatically reduced the abundances of pathogenic *Proteobacteria* and *Escherichia‐Shigella* in the gut (Figure 9l,m). DSS induction increased the numbers of *Proteobacteria* and *Escherichia‐Shigella* by 53% and 48% respectively, which were inhibited by 72% and 99% after EcN@PDNI treatment (Figure 9n,o). Meanwhile, *Mucispirillum*, which was responsible for repairing intestinal mucosa by colonization in colon,^[^ ^51^ ^]^ enriched remarkably by treating with EcN@PDNI (Figure 9p). Compared to DSS mice treated with PBS, the effect of EcN@PDNI toward the gut microbiota was significant, showing the ability to positively modulate the gut microbiota. As validated in Figure S18 in the Supporting Information, the improvement further ameliorated the intestinal permeability, which could be resulted from the combination effects of the modulated gut microbiota as well as the activated mucosal immunity. Briefly, EcN@PDNI could beneficially modulate the gut microbiota in IBD mice.

### 2.8 Treatment Efficacy of EcN@PDNI in IBD Mice Induced by Oxazolone

**Figure 10:**
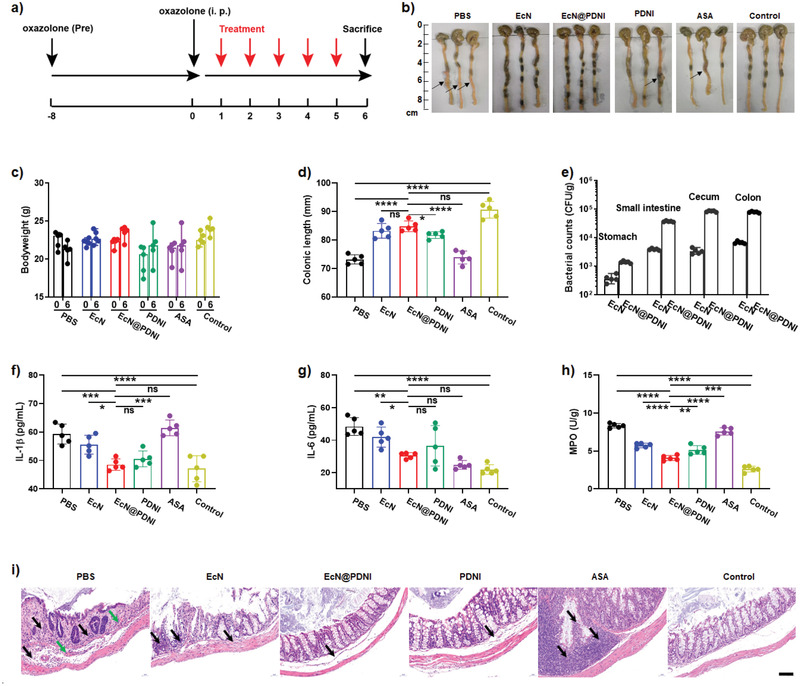
Therapeutic effect of EcN@PDNI in oxazolone mice. a) Experimental design for the treatment of a murine model of colitis induced by oxazolone. Mice were administrated with 1% oxazolone through anus following pretreatment with 3% oxazolone on their back. 0.2 mL PBS, EcN (2 × 10^8^ CFU), EcN@PDNI (2 × 10^8^ CFU), PDNI (10 mg kg^−1^), and ASA (60 mg kg^−1^) were orally dosed by gavage, respectively. All mice were euthanatized for collecting samples 5 d post‐treatment. Mice without oxazolone pretreatment were used as a control. b) Photographs of colons after treatment. Arrows indicate the inflammatory site. c) Bodyweights of mice at the indicated time points. d) Lengths of colons sampled from the treated mice. e) Counts of EcN reserved in the stomach, small intestine, cecum, and colon, respectively. Tissues were weighted and homogenized for bacterial plate counting. Concentrations of f) IL‐1*β* and g) IL‐6 in serum collected from the treated mice. h) Expression levels of MPO in colons after treatment. i) Representative H&E staining images of distal colons. Black and green arrows indicate inflammation and edema, respectively. Scale bar: 50 µm. Error bars represent standard error of mean (*n* = 5). *p* < 0.05, *, *p* < 0.01, **, *p* < 0.001, ***, *p* < 0.0001, ****. ns indicates no statistical significance.

By intrarectal administration of oxazolone (1%), we further built a murine model of colitis with a typical acute inflammation of distal colonic mucosa, characterized as epithelial damage and infiltration lymphocytes in mucosa.^[^ ^48^ ^]^ A similar treatment was conducted to evaluate the therapeutic effect of EcN@PDNI (**Figure** **10**a). Inflamed distal colonic mucosa appeared in mice dosed with PBS, PDNI, and ASA, whereas no obvious symptoms could be observed in EcN@PDNI treated mice (Figure 10b). Bodyweight of the mice recovered more efficiently during EcN@PDNI treatment (Figure 10c). The colonic length of EcN@PDNI treated mice was comparable to that of uninfected mice and closed to double long than those of mice administrated with PBS and ASA, respectively (Figure 10d). The delivered EcN@PDNI showed high level of colonization in the intestinal tract, which was near 10^5^ CFU per gram of the colon tissue (Figure 10e). Additionally, EcN@PDNI decreased the levels of MPO and inflammatory cytokines including IL‐1*β* and IL‐6 in contrast to PBS (Figure 10f–h). Importantly, in all treated mice, dosing with EcN@PDNI emerged the lowest concentrations of IL‐1*β* and MPO. The content of serotonin in serum of PDNI and EcN@PDNI treated mice showed the same level to other groups (Figure S19a, Supporting Information). Severe inflammation and edema appeared in the pathology of distal colon sectioned from mice treated with PBS as oxazolone could induce the injury of distal colon (Figure 10i). In comparison to other treated groups, EcN@PDNI dosed mice remitted the inflammation and edema effectively and displayed the lowest histopathology score (mean score 0.6) (Figure S19b, Supporting Information). Even comparing to clinical ASA, EcN@PDNI showed a higher activity in reducing inflammatory cytokines and repairing distal colonic mucosa.

In summary, we have described the use of dopaminergic immunoregulation to suppress hyperactive immune responses in local inflamed tissues. As a dopamine analogue, polydopamine nanoparticles have been prepared as an immunosuppressant and demonstrated its ability to involve in the dopaminergic system. PDNI can activate Treg cells, while directly inhibit Th cells including Th1, Th2, and Th17 cells. In addition to the direct intervention, PDNI prevents the activation of DCs which further upregulate the ratio of Treg/Th17. In a local inflamed tissue, PDNI has suppressed the immune overreaction and assisted the reversion of the inflammatory responses. We have further disclosed the application of dopaminergic immunoregulation by combining with gut microbiota modulation for treating IBD. The combination has been implemented by coating living beneficial bacteria with PDNI. Apart from the immunosuppression ability, PDNI protects the bacteria from environmental insults following oral delivery. Coated bacteria not only suppress the hyperactive immune responses but also positively modulate the gut microbiome in mice associated with colitis. Significantly, the combination of dopaminergic immunoregulation and gut microbiota modulation has demonstrated enhanced treatment efficacies in comparison with a clinical aminosalicylic acid in two murine models of colitis. To this end, future investigations are expected to evaluate biosafety issues and optimize dosage and treatment frequency, particularly in large animal models.

## Conflict of Interest

The authors declare no conflict of interest.

## Supporting information

Supporting Information

## Data Availability

The data that support the findings of this study are available from the corresponding author upon reasonable request.
