# Supplementary material for: Polydopamine Nanoparticle‐Mediated Dopaminergic Immunoregulation in Colitis
Source: Adv Sci (Weinh). 2021 Oct 28;9(1):2104006. doi: 10.1002/advs.202104006 (PMC8728836; doi:10.1002/advs.202104006)
Supplement: Supplementary file 1 — Supporting Information [file ADVS-9-2104006-s001.pdf]

ADVANCED SCIENCE
Open Access

Supporting Information

for *Adv. Sci.*, DOI: 10.1002/advs.202104006

Polydopamine nanoparticle-mediated dopaminergic immunoregulation in colitis

*Juanjuan Li, Weiliang Hou, Sisi Lin, Lu Wang, Chao Pan, Feng Wu, Jinyao Liu**

Supporting Information

Polydopamine nanoparticle-mediated dopaminergic immunoregulation in colitis

*Juanjuan Li#, Weiliang Hou#, Sisi Lin, Lu Wang, Chao Pan, Feng Wu, Jinyao Liu**

Experimental Section

Materials: EcN strain was purchased from China general microbiological culture collection center (GMCC, China) and grown in Luria Bertani (LB) medium at 37 °C with suitable antibiotics. DA, FITC, ASA, oxazolone, TNBS (5% in water), Dispase II, DNase I and Collagen IV were purchased from Sigma. $CD4^+$ T cell isolation kit were purchased from Metenyi Biotec. Percoll and Ficoll were obtained from GE Healthcare. Cell stimulation cocktail (plus protein transport inhibitors) (500 ×) was bought from ThermoFisher. Antibodies, containing anti-CD3 and anti-CD28, were bought from BD Bioscience. Antibodies including Percp-cy5.5 conjugated CD11b, FITC conjugated CD11c, APC conjugated anti-mouse CD86, PE conjugated anti-mouse MHC II, PE-cy7 conjugated anti-mouse CD25, FITC conjugated anti-mouse CD4, Percp-cy5.5 conjugated anti-mouse CD3, APC conjugated anti-mouse Foxp3, APC conjugated anti-mouse IL-4, PE-cy7 conjugated IFNγ, PE conjugated anti-mouse Foxp3 and PE conjugated anti-mouse IL-17A were received from Biolegend. DSS was obtained from Sangon Biotech (Shanghai, China). Tris-HCl buffer (pH 8.8, 10 mM) was purchased from Beyotime Biotechnology (Suzhou, China). Plasmids pBBR1MCS2-Tac-mCherry (Kanamycin resistant) and other reagents were obtained from domestic suppliers and used as received.

Cell culture

Primary $CD4^+$ lymphocytes were isolated from the spleen of C57BL/6J mice by using gradient centrifugation (Ficoll, GE Healthcare) and $CD4^+$ T cell isolation kit according to the operating instruction. Cells were cultured in RIPM 1640 (Gibco) supplemented with 10%

fetal bovine serum (Gibco), 100 U/ml of penicillin and 100 μg/ml of streptomycin (Gibco). BM-DCs were flushed out from the hind leg bones (femur and tibia) of C57BL/6J mice and cultured in RIPM1640 supplemented with 10% fetal bovine serum, 100 U/ml of penicillin, 100 μg/ml of streptomycin, and 20 ng/ml of granulocyte macrophage colony-stimulating factor (GM-CSF) for a week before use. Cells were cultured in a humidified atmosphere of 5% CO_2 at 37 °C. MODE-K and Caco-2 cells were cultured in high glucose DMEM supplemented 10% fetal bovine serum, 100 U/ml of penicillin and 100 μg/ml of streptomycin.

Animals

Balb/c mice (male, 6 weeks) and C57BL/6J (male, 6 weeks) were purchased from Jiesijie Laboratory Animal Technology. All the animal procedures complied with the guidelines of the Shanghai Medical Experimental Animal Care. Animal protocols were approved by the Institutional Animal Care and Use Committee of Shanghai Jiao Tong University School of Medicine.

Synthesis of PDNI and EcN@PDNI

PDNI was synthesized by self-polymerization of 1 mg/ml of DA in Tris-HCl (pH 8.8, 10 mM) for 0.5 hours and collected by centrifugation (15000 r/min). The obtained product was purified by washing with PBS for 3 times. To obtain fluorescent PDNI, FITC (1 mg/ml) or cy5.5 (1 mg/ml) was incubated with PDNI at room temperature for 2 hours and subsequently rinsed by water for several times. EcN@PDNI were prepared by shaking the bacteria in Tris-HCl buffer containing DA. Briefly, EcN were cultured in kanamycin containing LB medium for 4 hours and then washed with PBS. Afterward, 2×10^9 CFU of EcN were added into 3 ml of Tris-HCl buffer (pH 8.8, 1 mM) containing 1 mg/ml of DA and shaken for 0.5 hours at room temperature. EcN@PDNI were collected by centrifugation (7000 r/min) and purified by washing with PBS for 3 times.

Characterization of PDNI

To characterize the morphology of PDNI, 10 μl of dispersion was dipped on a 300-mesh copper formvar/carbon grid and rinsed twice by double distilled water, and the sample was observed under TEM (H7700s, Hitachi). The size distribution of PDNI was measured by DLS (Zetasizer Nano ZSP, Malvern). UV-Vis spectrum of PDNI was recorded by Shimadzu UV2550, and fluorescent spectra of PDNI-FITC and PDNI-cy5.5 were measured by Shimadzu RF 6000.

Characterization of EcN@PDNI

To characterize the morphology, 10 μl of the EcN@PDNI (2×10^7 CFU) suspension was loaded onto a 300-mesh copper formvar/carbon grid and rinsed twice by double distilled water. All samples were observed by TEM immediately after drying in air. For SEM (S-4800, Hitachi) observation, the bacteria were fixed in 2.5% glutaraldehyde solution for 2 hours at 4 °C. The resulted bacteria were rinsed by PBS for 3 times and serially dehydrated in 30%, 50%, 70%, 80%, 90% and 100% ethanol for 15 minutes. The dehydrated bacteria were added on silicon wafer, dried through natural desiccation and coated with the help of a sputtering coater before imaging. The size distribution and zeta potential of EcN and EcN@PDNI were measured by DLS. An amount of 1 ml of PBS containing 1×10^8 CFU bacteria was analyzed by DLS directly. mCherry expressing EcN were coated with FITC-labelled PDNI for flow cytometric analysis (LSRFortessa, BD Bioscience) and CLSM imaging (TCS-SP8 SR, Leica).

Biocompatibility evaluation of PDNI *in vitro* and *in vivo*

BM-DCs and MODE-K cells were seed in 96-well plate at a density of 1×10^4 cells, and cultured at 37 °C for 12 hours. Different concentrations of PDNI (128, 64, 32, 16, 8, 4 and 2 μg/ml) were added into the wells in three parallel, and incubated at 37 °C for 12 or 24 hours,

respectively. 100 μl 10% CCK8 was added into the plate after the cells were rinsed for 3 times with PBS, and OD_{450} was measured with a microplate reader after 45 minutes incubation. Additionally, MODE-K and Caco-2 cells were seeded in culture dishes with glass bottom at a density of 2×10^4 cells and permitted to grow at 37 °C for 12 hours. PDNI-FITC (100 μg/ml) was added into the dish, and then cells were rinsed after incubation for 2, 4, 8 and 24 hours, respectively. Cells were observed with CLSM, and then images were captured. Metabolism of PDNI *in vivo* was investigated in mice by administering PDNI-cy5.5, and then tracked with *in vivo* imaging system (INIS Lumina II, Caliper). Briefly, mice were administered with PDNI-cy5.5 (100 mg/kg) and then observed at different time point (2, 4, 8 and 24 hours post-administration). Fecal samples of mice were collected at same time point. Mice were euthanatized at 24 hours after gavage, and major organs including intestines, brain, lung, heart, liver, spleen and kidney were observed using *in vivo* imaging system. The sampled colon, liver and spleen tissues were fixed in 4% formalin, and performed H&E staining for pathology observation.

Immunomodulation of LPMC

Lymphocytes in the lamina propria were extracted according to the methods reported elsewhere.[1] Inflamed colon was developed by giving drinking water containing 3% DSS to Balb/c mice for 7 days. Mice without DSS water were used as a control. Mice were randomly divided into different groups and daily administrated with PBS and PDNI for 5 days, respectively. The bodyweight of mice was recorded daily and the mice were euthanatized after treatment. Additionally, $CD4^+$ lymphocytes dependent colitis induced by TNBS was built as a model for further understand the immunomodulation ability of PDNI. Balb/c mice were weighted and pretreated with a single dose of 100 μl 1% TNBS that was dissolved in a mixture of acetone and olive oil (acetone/olive oil = 4/1) on the back (1.5 × 1.5 cm). Mice

without TNBS pretreatment were used as a control. Mice were weighted at day 8 after pretreatment and excluded once the bodyweight decreased to 95% initial value. The rest mice were deposited with 100 μl 2.5% TNBS in 50% ethanol through anus using a 3.5-F catheter. Mice grouped in the control group were administrated with 100 μl 50% ethanol through anus. Mice with colitis were randomly divided into 3 groups and daily administrated with 0.2 ml PBS and PDNI (10 mg/kg) for 5 days, respectively. Pieces of colon (0.5 × 0.5 cm) from each mouse were sampled, cleaned with PBS, and shaken in digesting buffer (collagen IV, Dispase II and DNase I) at 37 °C for 40 minutes. Suspended cells were filtered by a 70 μm filter and collected by centrifugation (1000 g) for 10 minutes. The obtained cells were re-dispersed in 40% Percoll and then gently added into 70% Percoll. After centrifuging at 400 g for 25 minutes, cells located between the two liquid layers were collected and stimulated with PMA (10 ng/ml), ionomycin (1 μg/ml), and Brefeldin A (10 μg/ml) for 6 hours. One-third of the cells were stained with CD11c-FITC (1:200), CD11b-Percp-cy5.5 (1:500), CD86-APC (1:200) and MHC II-PE (1:200). Another one-third of the harvested cells were stained with anti-CD4-FITC and anti-CD25-PE-cy7 (1:200) for 45 minutes. The rest cells were stained with anti-CD3-Percp-cy5.5 and anti-CD4-FITC (1:200). After washing with PBS, the cells were fixed and permeated with FIX & PERM™ Cell Permeabilization Kit and then stained with anti-Foxp3-PE (1:200) or anti-IFNγ-PE-cy7/anti-IL-4-APC/anti-IL-17A-PE (1:200) for 2 hours, respectively. The stained cells were assayed by flow cytometer and 10000 relevant events were acquired for flow cytometric analysis. To measure the levels of IL-17A, TGFβ and IL-10, blood samples were collected and centrifuged at 4000 g for 5 minutes. The obtained serum samples were analyzed by using ELISA kit.

Activation of DCs and T lymphocytes by PDNI

2×10^5 of bone marrow cells were cultivated in 1 ml medium containing GM-CSF (20 ng/ml) in a 24-well plate for a week to produce BM-DCs. PDNI with different concentrations (50, 20, 10 and 5 μg/ml) was added into the cells and cultured for 12 hours. Cells were harvested and washed for 3 times, and then incubated with anti-CD86-APC and anti-MHC II-PE at 37 °C for 45 minutes. Cells were analyzed by flow cytometer after rinsing with PBS for several times. 1×10^5 $CD4^+$ lymphocytes extracted from C57BL/6J mice were cocultured with anti-CD3&CD28 (2.5 μg/ml&2.0 μg/ml), anti-CD3&CD28&TGFβ (2.5 μg/ml&2.0 μg/ml&2.5 ng/ml), and anti-CD3&CD28&TGFβ/PDNI (2.5 μg/ml&2.0 μg/ml&2.5 ng/ml/20 μg/ml) in medium supplied with IL-2 (100 U/ml) in 96-well plate, respectively. After incubation for 5 days, cells were harvested and washed with PBS for 3 times by centrifugation. The obtained cells were stained and assayed by flow cytometer as described above. To evaluate the activation of DCs, 2×10^5 of bone marrow cells were cultivated in 1 ml medium containing GM-CSF in a 24-well plate for a week to produce BM-DCs. After washing with PBS, BM-DCs were treated with PBS, LPS (1 μg) and LPS/PDNI (1/20 μg) in 1 ml medium for 12 hours at 37 °C. Media of BM-DCs were collected for the analysis of IL-10, IL-1β, IL-6 and TNFα by ELISA kits. Meanwhile, cells were harvested and collected after washing with PBS for 3 times. The obtained cells were stained with CD86-APC (1:200) and MHC II-PE (1:200) antibodies for 45 minutes at 37 °C. After rinsing with PBS for 3 times, the stained cells were analyzed by flow cytometer. To further activate lymphocytes, 5×10^5 $CD4^+$ lymphocytes were added into the plate containing pretreated 1×10^5 BM-DCs. The lymphocytes were collected after incubation for 5 days at 37 °C and stained for flow cytometric analysis.

***In vitro* bacterial viability**

1×10^5 CFU EcN or EcN@PDNI were added into 1 ml medium including SGF containing 10 g/l pepsin in 0.85% NaCl solution (HCl, pH 1.8), 0.3 mg/ml CA in PBS (pH 7.4), and SIF

containing 10 g/l trypsin in KH_2PO_4 solution (NaOH, pH 6.8), respectively. After incubation at 37 °C for 0.5, 1, 2, 3 and 4 hours, the bacteria were harvested by centrifugation and re-suspended in 1 ml PBS. 100 μl bacterial suspension was spread onto ager plate and incubated at 37 °C for 24 hours before plate counting. 10 μl EcN@PDNI suspension after incubation for 2 and 4 hours were added onto a 300-mesh copper formvar/carbon grid, washed with double distilled water and observed by TEM after drying in air.

Stability of PDNI coating

To measure the stability of PDNI coating, 1×10^5 CFU EcN coated with FITC-labelled PDNI were cultured in 1 ml SIF for different time intervals. Afterward, the incubated bacteria were sampled for flow cytometric and CLSM imaging analysis. Excitations of FITC and EcN expressing mCherry were set at 488 and 562 nm, respectively. Additionally, the motilities of EcN and EcN@PDNI were measured by using semi solid medium. Berifly, bacterial dispersions were penetrated into semi solid medium with a fine needle, and then incubated at 37 °C for 24 hours.

***In vivo* bacterial survival**

DSS mice were administrated with 2×10^8 CFU EcN or EcN@PDNI by oral gavage and imaged by *in vivo* imaging system 4 hours post-administration. The images of the mice were recorded and analyzed by Living Image 4.2. The GI tract tissues were harvested and also observed by *in vivo* imaging system. Fluorescent signals were calculated for each part of the GI tract, including the stomach, small intestine, cecum and colon. To quantify the numbers of the survived EcN in the GI tract, given amounts of the tissues and their associated contents were collected and homogenized in 1 ml PBS. 100 μl of each dispersion was spread onto ager plate and cultured at 37 °C for 24 hours before counting. Furthermore, fecal samples of mice

at 1, 2, 4, 8, 12 and 24 hours post administration of EcN@PDNI were collected to measure the concectration of PDNI.

Cellular uptake of EcN@PDNI

Caco-2 cells were seeded in culture dishes with glass bottom at a density of 2×10^4 cells and permitted to grow at 37 °C for 12 hours. EcN@PDNI-FITC (10^8 CFU) were added into the dishes and cells were rinsed after incubation for 2, 4, 8 and 24 hours, respectively. Cells were observed with CLSM and images were captured.

Treatment of DSS mice

Balb/c mice were pretreated with 3% DSS drinking water for a week to develop colitis. Mice without pretreatment were used as a control. Mice with colitis were randomly divided into 5 groups and daily administrated with 0.2 ml PBS, EcN (2×10^8 CFU), EcN@PDNI (2×10^8 CFU), PDNI (10 mg/kg) and ASA (60 mg/kg) for 5 days, respectively. Mice were weighted and the pattern of stool was recorded during the experiment every other day and euthanatized by asphyxia with CO_2. Blood and colon of the mice were sampled for blinded histopathology analysis. Serum samples were obtained by centrifugation at 4000 g for 5 minutes. The concentrations of IL-1β, IL-6 and serotonin were detected by ELISA kits.

Treatment of oxazolone mice

A murine model of colitis was built by oxazolone induction according to the methods reported elsewhere[2]. Balb/c mice were weighted and pretreated with a single dose of 100 μl 3% oxazolone that dissolved in a mixture of acetone and olive oil (acetone/olive oil = 4/1) on the back (1.5 × 1.5 cm). Mice without oxazolone pretreatment were used as a control. Mice were weighted at day 8 after pretreatment and excluded once the bodyweight decreased to 95% the

initial values. The rest mice were deposited with 100 μl 1% oxazolone in 50% ethanol through anus using a 3.5-F catheter. Mice grouped in the control group were administrated with 100 μl 50% ethanol through anus. Mice with colitis were randomly divided into 5 groups and daily administrated with 0.2 ml PBS, EcN (2×10^8 CFU), EcN@PDNI (2×10^8 CFU), PDNI (10 mg/kg) and ASA (60 mg/kg) for 5 days, respectively. After treatment, the mice were euthanatized by asphyxia with CO_2. Both blood and colon of the mice were collected for blinded histopathology analysis. Serum samples were isolated from blood by centrifugation at 4000 g for 5 minutes. The levels of IL-1β, IL-6 and serotonin were assessed by ELISA kits.

Histopathologic analysis

Inflamed colons sectioned from the treated mice were fixed in 4% formalin and processed according to standard procedures for paraffin embedding. A sample with 4 μm thickness was sliced and stained with H&E. The obtained tissues were scanned by 3D HISTECH Pannoramic 250 (3DHISTECH, Hungary). Each section was evaluated for the presences of mono-nuclear cell infiltration, polymorphonuclear cell infiltration, epithelial hyperplasia and epithelial injury. Histopathology scores were independently graded as absent (0), mild (1), moderate (2), severe (3), giving a total score of 0 to 12.

MPO detection

Samples of the inflamed colon (0.5 × 0.5 cm) were weighted and immersed in 1 ml PBS. The rinsed samples were homogenized by a tissue homogenizer at 5000 r/min. The supernatants were collected by centrifugation at 1000 g for 5 minutes. The levels of MPO in the obtained samples were measured by ELISA kits.

DNA extraction and 16s sequencing and analysis

The 16s gene sequencing and analysis were conducted at Biomarker Technologies (Beijing, China). The total DNA was extracted form colonic content and sequenced by building sequencing library on Illumina HiSeq 2500. The original image data files obtained by high-throughput sequencing were converted into Sequenced Reads by Base Calling analysis. The results were stored in FASTQ (referred to as fq) format file, which contains sequence information of reads and their corresponding sequencing quality information. Data analysis was performed on BMKCloud. ACE index and Shannon index were analyzed to study the species diversity within a single sample. PCA and PCoA plots were conducted to compare the differences of species diversity between different samples. Taxonomic analysis was performed on samples at various taxonomic levels to obtain the community structure at the taxonomic level of phylum and genus.

Data analysis

Three independent experiments in the cell culture and at least five independent experiments in animal studies were conducted in this research. Statistical analysis was performed by using Excel and IBM SPSS. Data was analyzed by SPSS software and samples between groups were compared with ANOVA. Multiple comparisons were made by least-significant difference (LSD) method.

References

1. I. I. Ivanov, B. S. McKenzie, L. Zhou, C. E. Tadokoro, A. Lepelley, J. J. Lafaille, D. J. Cua, D. R. Littman, *Cell* **2006**, *126*, 1121-1133.
2. S. Wirtz, V. Popp, M. Kindermann, K. Gerlach, B. Weigmann, S. Fichtner-Feigl, M. F. Neurath, *Nat Protoc* **2017**, *12*, 1295-1309.

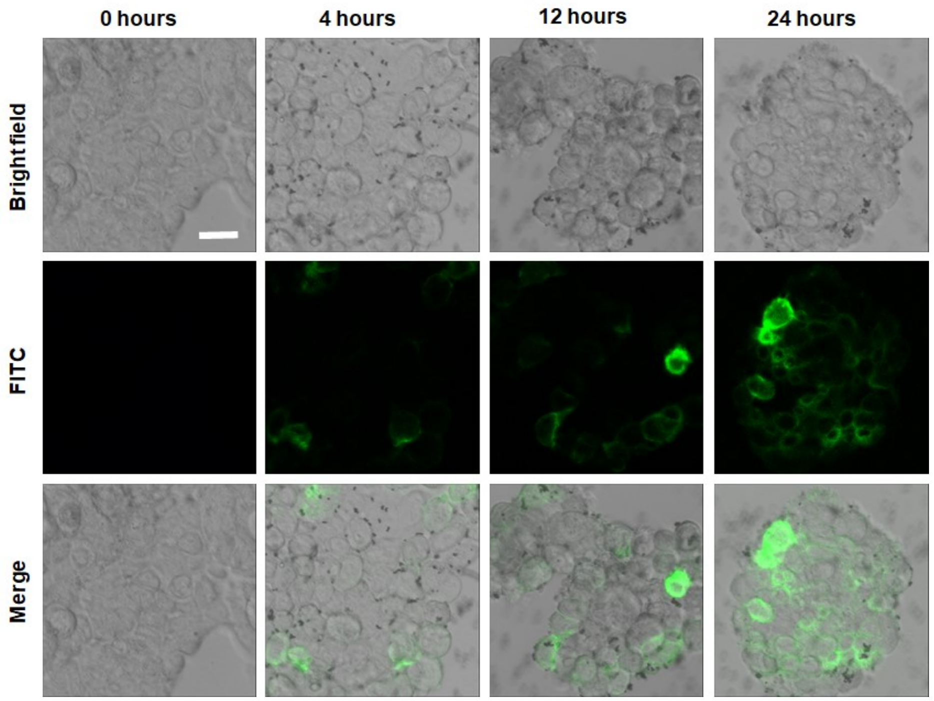

Figure S1 CLSM images of Caco-2 cells after incubation with PDNI-FITC (100 μg/ml) for different time intervals. Scale bar: 20 μm.

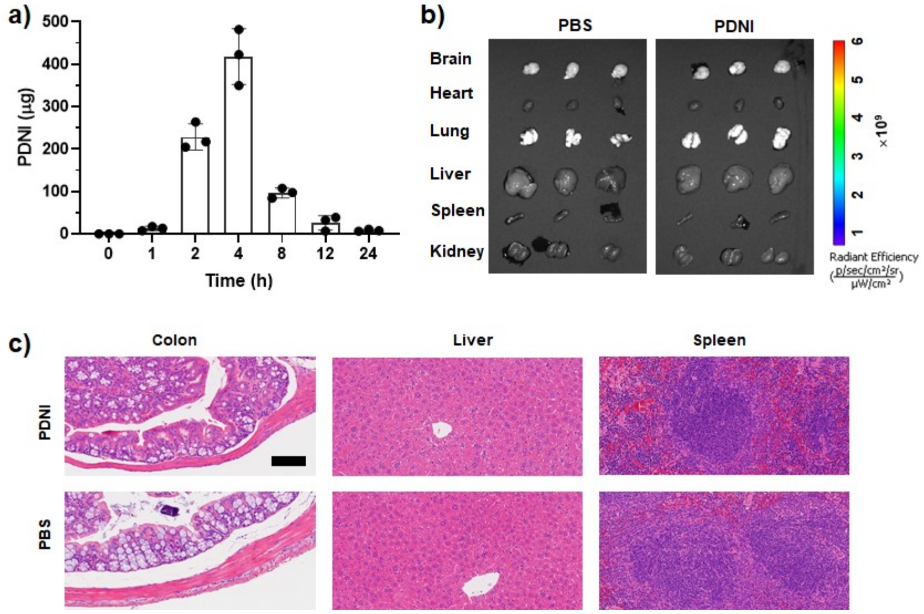

Figure S2 Toxicity evaluation of PDNI *in vivo*. **a** Contents of PDNI in the fecal samples of mice orally administered with PDNI (100 mg/kg bodyweight) at 1, 2, 4, 8, 12 and 24 hours post oral gavage. **b** *In vivo* imaging system measurement of major organs sectioned from mice orally administered with PDNI at 24 hours post-gavage. **c** Typical H&E staining of the colon, liver and spleen tissues. Scale bar: 100 μm.

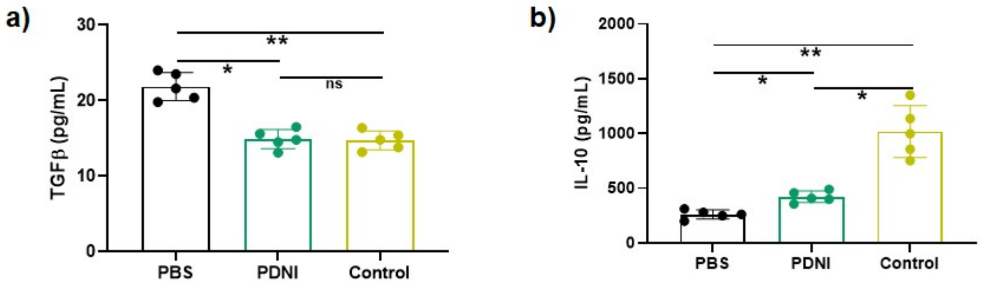

Figure S3 Levels of (**a**) TGFβ and (**b**) IL-10 in serum sampled from mice in the control, PBS and PDNI groups, respectively. Mice were pretreated with 3% DSS drinking water for a week and then orally dosed with PDNI (10 mg/kg) by gavage. Mice without pretreatment were used as a control. Error bars represent standard error of mean (n = 5). $p < 0.05$, *, $p < 0.01$, **. ns indicates no statistical significance.

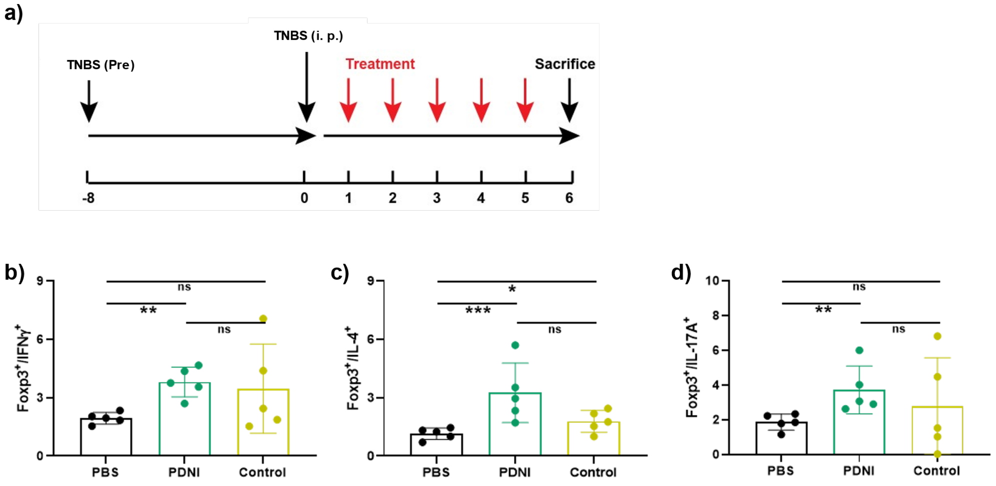

Figure S4 Immunomodulation activity of PDNI in the inflamed tissue. **a** Mice were administered with 2.5% TNBS through anus following pretreatment with 1% TNBS on their back. 0.2 ml PBS and PDNI (10 mg/kg) was orally dosed by gavage, respectively. All mice were euthanatized for collecting samples 5 days post-treatment. Mice without TNBS pretreatment were used as a control. LPMC were harvested from the inflamed colon 5 days post-administration and immediately marked with corresponding antibodies. **b-d** Ratios of (**b**) $CD4^+CD25^+Foxp3^+/CD4^+IFN\gamma^+$, (**c**) $CD4^+CD25^+Foxp3^+/CD4^+IL\text{-}4$ and (**d**) $CD4^+CD25^+Foxp3^+/CD4^+IL\text{-}17A^+$ in LPMC. Error bars represent standard error of mean (n = 5). $p < 0.05$, *, $p < 0.01$, **, $p < 0.001$, ***. ns indicates no statistical significance.

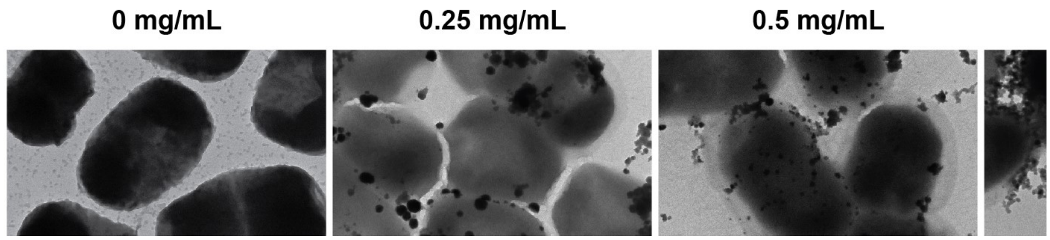

Figure S5 TEM images of EcN after incubation with different concentrations of DA. Scale bar: 1 μm.

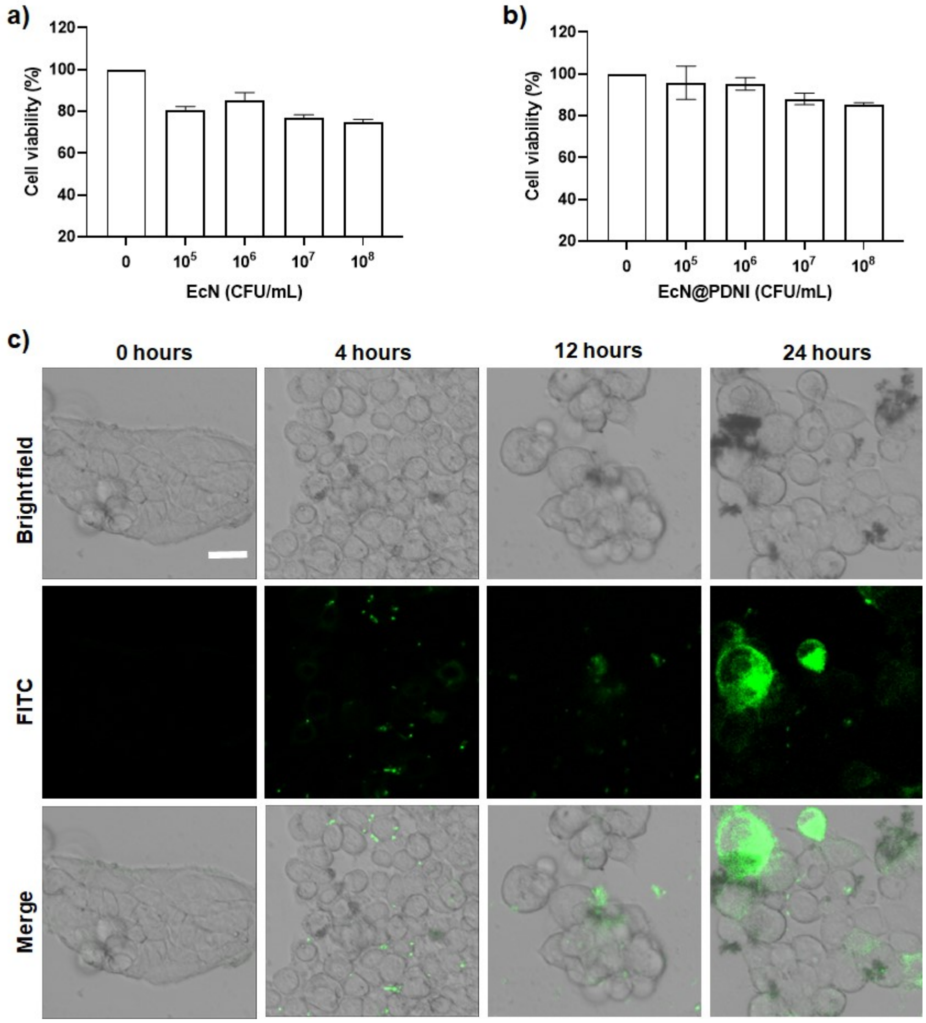

Figure S6 Cytotoxicity and cellular uptake of EcN@PDNI. **a**, **b** Cell viability of MODE-K cells after incubation with (**a**) EcN and (**b**) EcN@PDNI for 24 hours, respectively. **c** CLSM images of Caco-2 cells after incubation with EcN@PDNI for different time intervals. Scale bar: 20 µm.

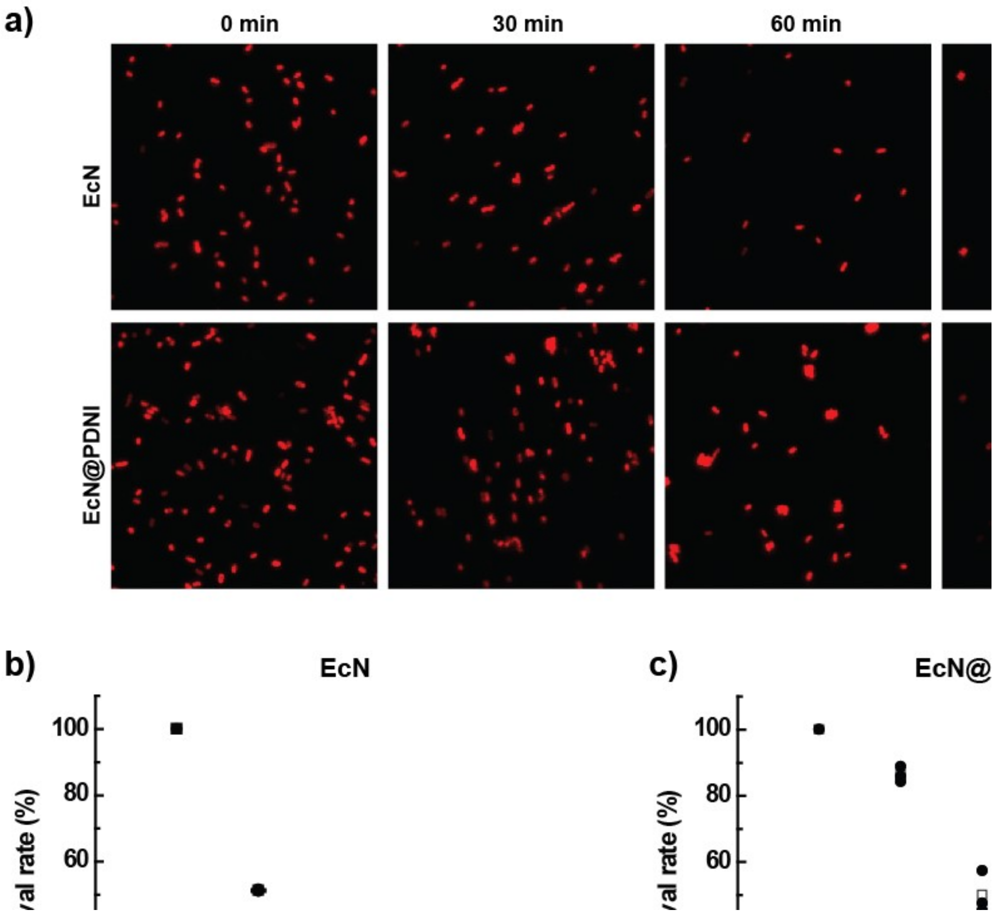

Figure S7 a CLSM images of bacteria after incubation with SGF. Scale bar: 10 μm. **b**, **c** Survival rates of (**b**) EcN and (**c**) EcN@PDNI in SGF. Error bars represent standard error of mean ($n = 3$).

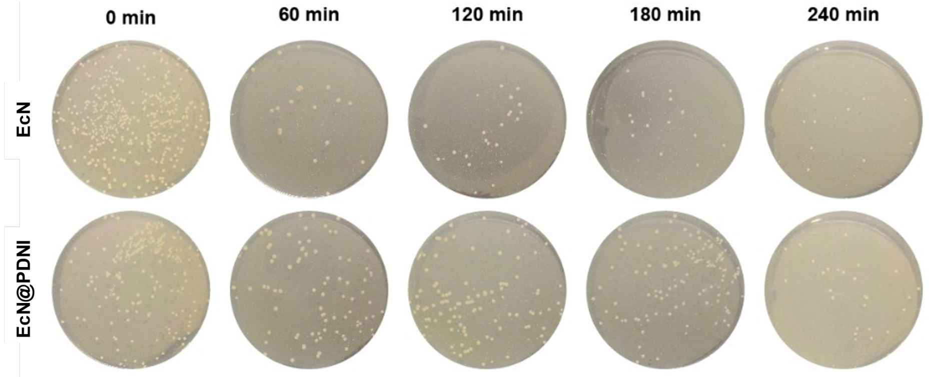

Figure S8 Plates of EcN and EcN@PDNI after incubation in SGF for 0, 60, 120, 180 and 240 minutes, respectively. 100 µl of dispersions containing bacteria were spread onto antibiotic containing agar plate and incubated at 37 °C for 24 hours.

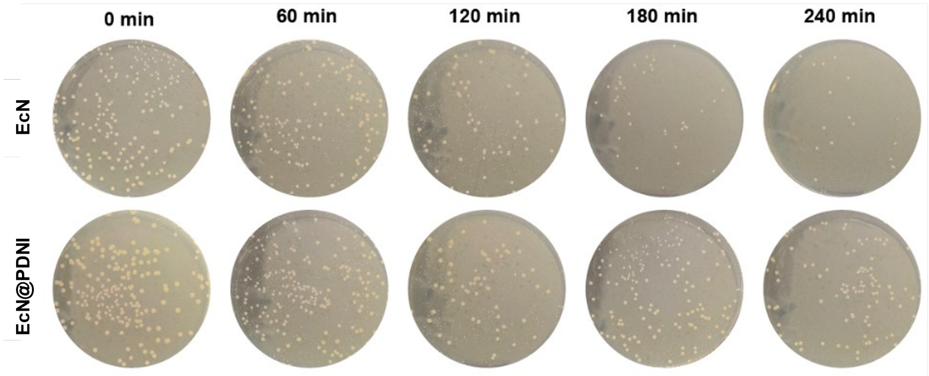

Figure S9 Plates of EcN and EcN@PDNI after incubation in CA for 0, 60, 120, 180 and 240 minutes, respectively. 100 µl of dispersions containing bacteria were spread onto antibiotic containing agar plate and incubated at 37 °C for 24 hours.

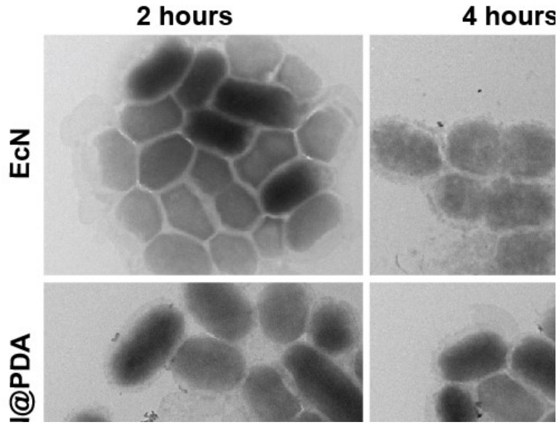

Figure S10 TEM images of EcN and EcN@PDNI after incubation in SGF for 2 and 4 hours, respectively. Scale bar: 2 μm.

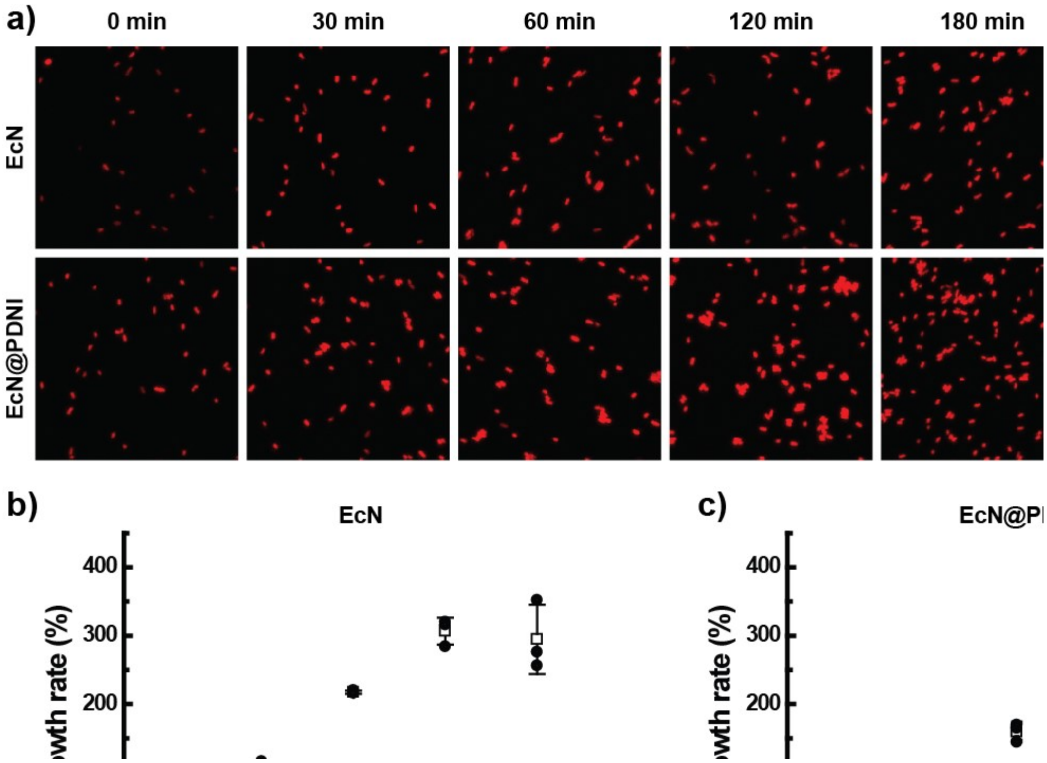

Figure S11 **a** CLSM images of EcN and EcN@PDNI after incubation in SIF for the indicated time points. Scale bar: 10 μm. **b**, **c** Growth profiles of (**b**) EcN and (**c**) EcN@PDNI in SIF. Error bars represent standard error of mean (n = 3).

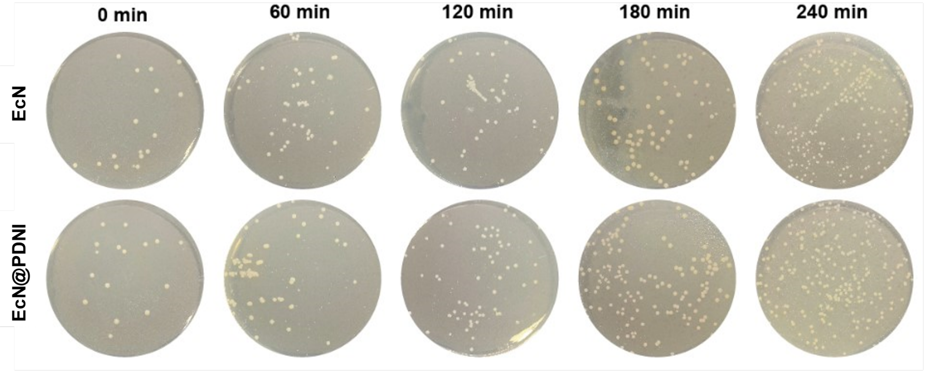

Figure S12 Plates of EcN and EcN@PDNI after incubation in SIF for 0, 60, 120, 180 and 240 minutes, respectively. 100 μl of dispersions containing bacteria were spread onto antibiotic containing agar plate and incubated at 37 °C for 24 hours.

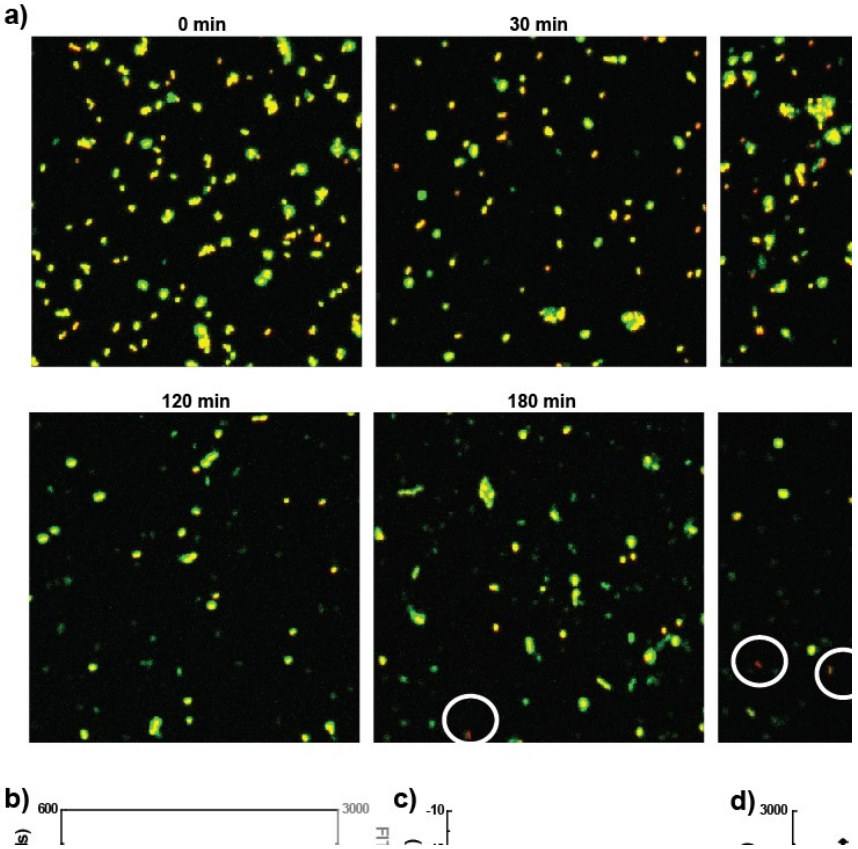

Figure S13 **a** CLSM images of EcN@PDNI after incubation in SIF for the indicated time points, and the white circles indicate progeny of EcN@PDNI. Scale bar: 10 μm. **b** Variation of fluorescence intensity of EcN coated with FITC-labelled PDNI in SIF. **c**, **d** Variations of (**c**) zeta potential and (**d**) size distribution of EcN@PDNI during incubation in SIF. Error bars represent standard error of mean (*n* = 3).

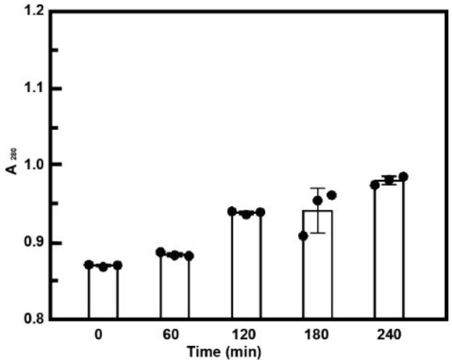

Figure S14 Absorbance of the supernatant of EcN@PDNI at 280 nm after incubation in SIF for the indicated time points. Error bars represent standard error of mean (n = 3).

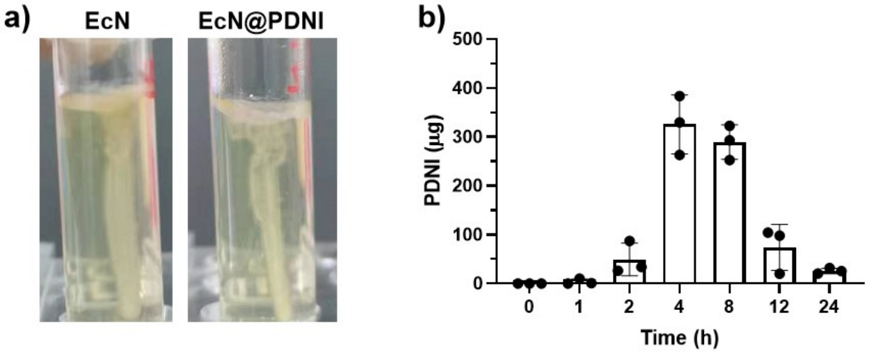

Figure S15 a Motilities of EcN and EcN@PDNI measured by determining the distribution of bacteria throughout the capillary tube. **b** Contents of PDNI in the fecal samples of mice orally delivered with EcN@PDNI (10^9 CFU) at 1, 2, 4, 8, 12 and 24 hours post oral gavage.

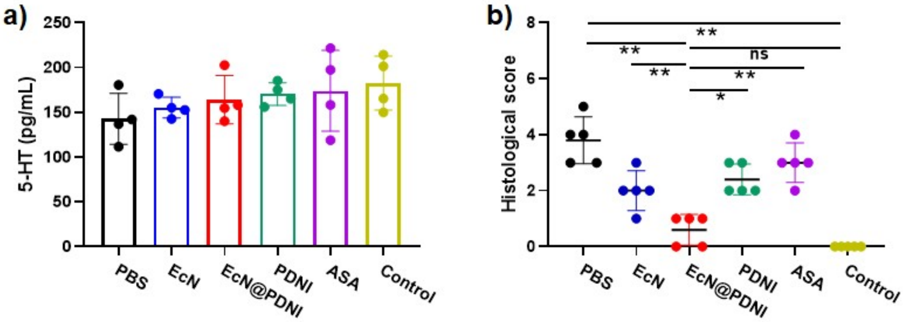

Figure S16 a Concentrations of serotonin in serum samples of mice treated with PBS, EcN, EcN@PDNI, PDNI and ASA, respectively. **b** Histopathology scores of proximal colons after treatment with PBS, EcN, EcN@PDNI, PDNI and ASA, respectively. Mice were fed with 3% DSS for 7 days and then treated with PBS, EcN, EcN@PDNI, PDNI and ASA for 5 days (n = 5). $p < 0.05$, *, $p < 0.01$, **. ns indicates no statistical significance.

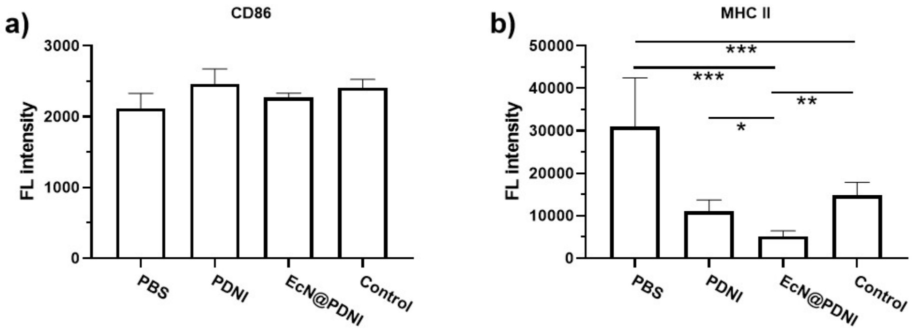

Figure S17 Effects of PDNI and EcN@PDNI on DCs in DSS-induced mice. Expresion levels of (**a**) CD86 and (**b**) MHC II on DCs. $p < 0.05$, *, $p < 0.01$, **, $p < 0.001$, ***.

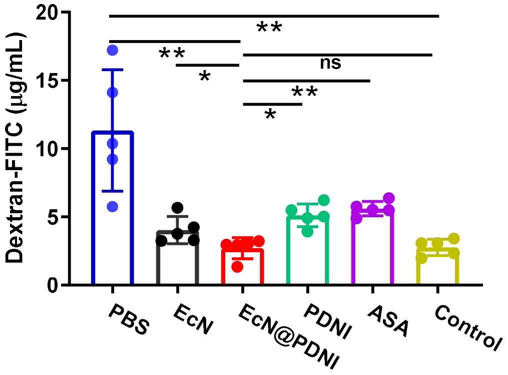

Figure S18 Intestinal permeability of mice after treatment. Fluorescence intensity of serum from DSS mice was measured after gavage of 60 mg/kg FITC conjugated dextran. Error bars represent standard error of mean ($n = 5$). $p < 0.05$, *, $p < 0.01$, **. ns indicates no statistical significance.

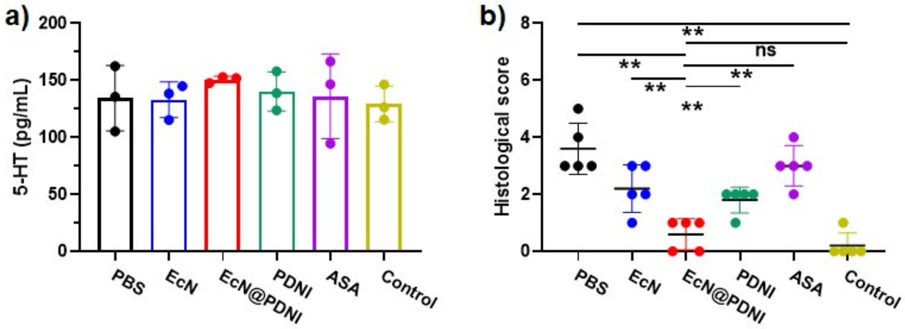

Figure S19 a Concentrations of serotonin in serum samples of mice after treatment with PBS, EcN, EcN@PDNI, PDNI and ASA, respectively. **b** Histopathology scores of distal colons after treatment with PBS, EcN, EcN@PDNI, PDNI and ASA, respectively. Mice were administered with 1% oxazolone through anus following pretreatment with 3% oxazolone on their back, and then treated with PBS, EcN, EcN@PDNI, PDNI and ASA for 5 days ($n = 5$). $p < 0.01$, **. ns indicates no statistical significance.
